# Genarris 3.0: Generating Close-Packed Molecular Crystal Structures with Rigid Press

**DOI:** 10.1021/acs.jctc.5c01080

**Published:** 2025-10-30

**Authors:** Yi Yang, Rithwik Tom, Jose A. G. L. Wui, Jonathan E. Moussa, Noa Marom

**Affiliations:** † Department of Materials Science & Engineering, 6612 Carnegie Mellon University, Pittsburgh, Pennsylvania 15213, United States; ‡ Department of Physics, 6612 Carnegie Mellon University, Pittsburgh, Pennsylvania 15213, United States; § Department of Physics, University of Texas at Austin, Austin, Texas 78712, United States; ∥ Molecular Sciences Software Institute, 1757 Virginia Tech, Blacksburg, Virginia 24060, United States; ⊥ Department of Chemistry, 6612 Carnegie Mellon University, Pittsburgh, Pennsylvania 15213, United States

## Abstract

Polymorphism in molecular crystals influences their properties and performance. Crystal structure prediction (CSP) can help explore the crystal structure landscape and discover potentially stable polymorphs computationally. We present a new version of the Genarris open-source code, which generates random molecular crystal structures in all space groups and applies physical constraints on intermolecular distances. The main new feature in Genarris 3.0 is the “Rigid Press” algorithm, which uses a regularized hard-sphere potential to compress the unit cell and achieve a maximally close-packed structure based on purely geometric considerations without performing any energy evaluations. In addition, Genarris 3.0 is interfaced with machine-learned interatomic potentials (MLIPs) to accelerate the exploration of the potential energy landscape. We present a new clustering and down-selection workflow that employs the MACE-OFF23­(L) MLIPs to perform geometry optimization and energy ranking in the early stages. We use Genarris 3.0 to successfully predict the structure of six targets: aspirin, Target I and Target XXII from previous CSP blind tests, and the energetic materials HMX, CL-20, and DNI. We further analyze the performance of MACE-OFF23­(L) compared to dispersion-inclusive density functional theory (DFT) for geometry relaxation and energy ranking. We find significant variability in the performance of MACE-OFF23­(L) across chemically diverse targets with particularly poor performance for energetic materials, which is mitigated by our clustering and down-selection procedure. Genarris 3.0 can thus be used effectively to perform CSP and to generate molecular crystal data sets for training ML models.

## Graphical abstract

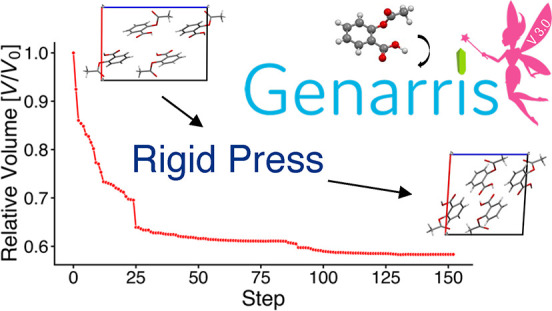

## Introduction

Molecular crystals are used for diverse applications including organic semiconductor devices,^1^ energetic materials (EMs),^2,3^ pharmaceuticals,^4^ and agricultural chemicals.^5^ Because molecular crystals are held together by weak van der Waals interactions, they are prone to polymorphism,^6^ which is the ability of the same compound to crystallize into multiple crystal structures. Polymorphism has a far-reaching impact because different polymorphs can have markedly different physical, chemical, and mechanical properties. For example, crystal structure can influence the bioavailability and stability of pharmaceuticals,^7,8^ the sensitivity, detonation velocity, and safety of energetic materials,^3,9–11^ and the charge carrier mobility of organic semiconductors.^12,13^ Consequently, a comprehensive understanding of crystal structure landscapes and screening for polymorphs with desired properties is essential for the development of products based on molecular crystals. It can be time-consuming to perform exhaustive polymorph screening because minor variations in crystallization conditions can alter the resulting crystal structure and some structures are difficult to crystallize.^14–17^ Computer simulations can provide guidance as to the possible presence of thermodynamically stable polymorphs, which have not yet been experimentally obtained. Indeed, computational crystal structure prediction (CSP) has become an integral part of the pharmaceutical development pipeline.^18–21^ Moreover, computer simulations can further predict the properties of putative crystal structures.^22–29^

Computational CSP aims to predict all plausible polymorphs of a given compound. Advancements in CSP have been tracked through a series of blind tests organized by the Cambridge Crystallographic Data Centre (CCDC).^30–37^ The CSP blind tests have both benchmarked and driven methodological improvements. In addition, they have highlighted the challenges faced by state-of-the-art CSP methods. Over the years, as CSP capabilities have evolved, the complexity of the target systems has increased. The field has progressed from relatively rigid small molecules to more flexible, larger molecules, and from single-component to multicomponent crystals. As CSP targets become more complex, the configuration space that needs to be explored grows exponentially.^38–40^ This may require evaluating the relative stability of millions of putative structures. The difficulty is compounded by the fact that the energy differences between polymorphs are usually only a few kJ/mol,^41,42^ requiring high accuracy. The necessary accuracy can be achieved by dispersion-inclusive density functional theory (DFT),^43–56^ albeit at a high computational cost. Some intertwined challenges the CSP community is still grappling with are predicting stability at finite temperatures,^53,57–59^ the so-called overprediction problem, where structures corresponding to distinct local minima at 0 K correspond to the same (possibly disordered) structure at finite temperatures,^60^ and crystallographic disorder, caused by multiple molecular conformations, orientations, or atomic positions within the unit cell.^36^ Addressing these challenges would require going beyond lattice energy evaluations using dispersion-inclusive DFT at 0 K. This calls for the development of ranking methods that are both cost-effective and accurate for optimizing and evaluating the relative lattice energies of millions of candidate crystal structures.

Machine learned interatomic potentials (MLIPs) are considered as a promising route for achieving comparable accuracy to DFT at a significantly lower computational cost.^61–68^ To this end, MLIPs must be trained on large DFT data sets. Most of the available materials data sets are either of inorganic crystals with relatively small unit cells^62,69–73^ or of isolated small organic molecules.^74–81^ MLIPs have limited transferability outside of their training domains.^82^ The lag in the development of MLIPs for molecular crystals may therefore be attributed to the dearth of open data sets for molecular crystals. In order to perform well for molecular crystals, MLIPs must adequately capture intermolecular dispersion interactions. An alternative approach to training directly on molecular crystals is training on molecular data sets that include intramolecular dispersion interactions and/or intermolecular interactions between clusters of molecules. The resulting MLIPs, which capture short-range interactions, are then augmented with dispersion corrections, similar to DFT functionals.^67,83–86^

The 7^th^ CSP blind test was conducted in two phases, which ran from October 2020 to June 2022. The structure generation phase tested the ability of participants to generate the experimentally observed crystal structure starting from a molecular “stick diagram”.^36^ The ranking phase tested the ability of participants to relax and rank lists of structures provided by the CCDC.^37^ Our team (Group 16) used Genarris^87,88^ for crystal structure generation and system-specific AIMNet2 ^67,89^ MLIPs for geometry relaxation and energy ranking. Random or quasi-random crystal structure generation methods are frequently employed in CSP workflows to explore the potential energy surfaces (PES) of complex molecules with an unbiased sampling of crystal packing.^90–93^ Genarris generates random structures in all space groups compatible with the molecular symmetry and the requested number of molecules per unit cell (*Z*), including molecules occupying special Wyckoff positions. The target unit cell volume is determined by a machine-learned model^94^ and physical constraints are imposed on the intermolecular distances. The version of Genarris that was used in the 7^th^ CSP blind test employed a preliminary implementation of the Rigid Press algorithm, described below, which uses a regularized hard-sphere potential to achieve close packing of molecules in the unit cell. In the structure generation phase, system-specific AIMNet2 potentials were used to relax and rank millions of structures generated by Genarris. To the best of our knowledge, this was the earliest use (in 2020–2021) of MLIPs for molecular crystal structure prediction. We successfully generated four out of the six possible crystal structures for the targets we attempted, resulting in a success rate of 67%, which was the highest among academic teams and third overall.^36^ In the ranking phase, our system-specific AIMNet2 potentials attained accuracy on par with dispersion-inclusive DFT methods at a fraction of the computational cost, and exceeded the performance of the MLIPs used by two other teams (Groups 12 and 15).^37^ A detailed description of the system-specific AIMNet2 potentials and analysis of our results from the 7^th^ CSP blind test is provided elsewhere.^89^ Since the conclusion of the 7^th^ CSP blind test, others have reported incorporating MLIPs for structure optimization and energy ranking in CSP workflows.^95–97^ Generative models^98,99^ and large language models (LLMs)^100,101^ are emerging as promising future approaches to structure generation.

Here, we introduce Genarris 3.0, the latest version of our open-source Python package for molecular crystal structure generation. We provide a detailed description of the Rigid Press algorithm featured in this version. Genarris 3.0 is interfaced with a variety of energy evaluation and relaxation methods via the Atomic Simulation Environment (ASE),^102^ providing the user maximal flexibility for choosing their preferred methods. Here, the MACE-OFF^61^ MLIPs are employed to accelerate energy evaluations and geometry relaxations. A new workflow for down-selection is presented to gradually reduce the number of candidate structures evaluated with increasingly computationally expensive and more accurate methods. The modular and extensible design of Genarris facilitates the integration of advanced methods for structure generation, optimization, and energy evaluations, as well as the implementation of user-defined workflows, thereby enhancing its capabilities in CSP.

To demonstrate the performance of Genarris 3.0, we have selected six diverse targets, shown in Figure 1. Aspirin (2-acetoxybenzoic acid) is a representative example of a hydrogen bonded crystal. It has two polymorphs, Form I and Form II (CSD reference codes ACSALA and ACSALA17).^103,104^ Both forms have four molecules per unit cell (*Z* = 4) and crystallize in the monoclinic space group *P*2_1_/*c* (No. 14). Target I from the first CSP blind test (3,4-cyclobutylfuran)^30^ has no strong intermolecular interactions. It has two known polymorphs: a stable form that crystallizes in the monoclinic space group *P*2_1_/*c* (No. 14) with *Z* = 4 and a metastable form that crystallizes in the orthorhombic space group *Pbca* (No. 61) with *Z* = 8 (CSD reference codes XULDUD01 and XULDUD). Here, we focus on the structure with *Z* = 8, because its higher complexity and larger unit cell size provide a stringent test case for demonstrating the capability of our method to generate crystal structures with higher molecular packing complexity. Target XXII (tricyano-1,4-dithiino­[*c*]-isothiazole) from the sixth CSP blind test^35^ has unusual intermolecular interactions involving C, S, and N atoms. It crystallizes in the monoclinic space group *P*2_1_/*n* (No. 14) with *Z* = 4 (CSD reference code NACJAF).

**Figure 1:**
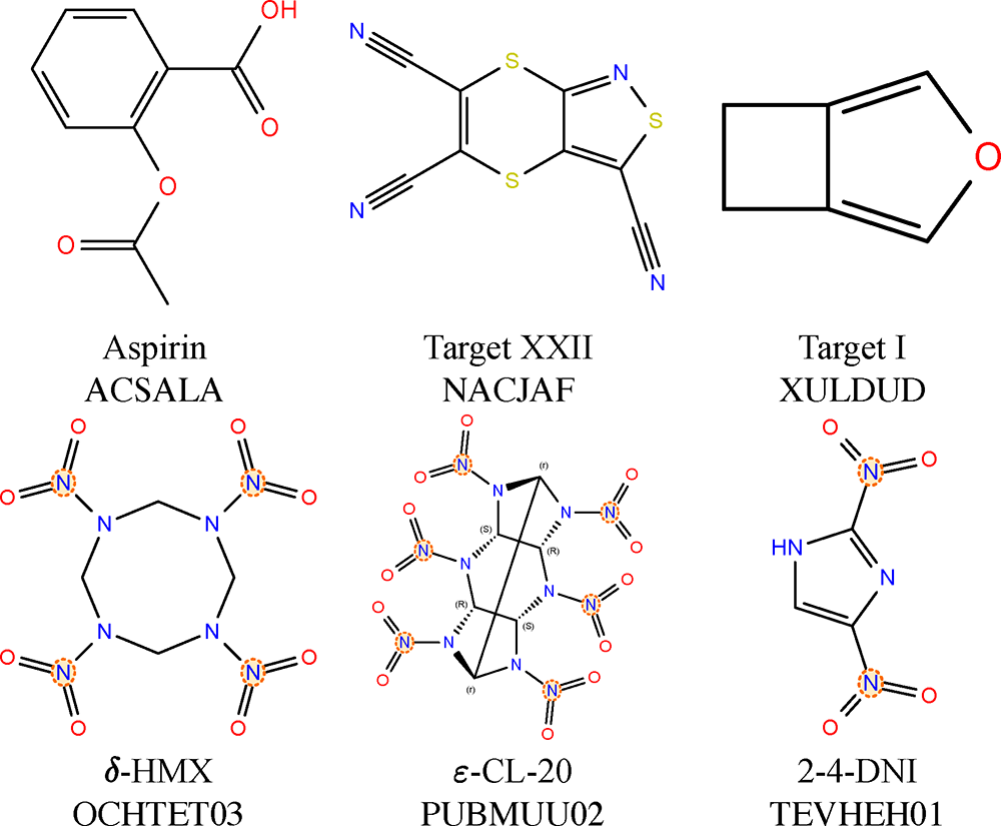
2D molecular diagrams, common names, and CSD reference codes of the six CSP targets used here.

In addition, we have selected three energetic materials (EMs). EMs are characterized by exceptionally dense crystal structures and strong intermolecular interactions between nitrogen-containing moieties.^54^ Given that experiments on EMs are inherently risky, CSP represents a valuable approach for safely and effectively exploring their landscapes.^10,11,105^ CL-20 (2,4,6,8,10,12-Hexanitro-2,4,6,8,10,12-hexaazatetracyclo­[5.5.0.0^3,11^.0^5,9^]­dodecane) has several known polymorphs.^106–108^ Here, we focus on the most stable form, ε-CL-20 (CSD reference code PUBMUU02), which possesses the highest density, greatest detonation velocity, and superior impact stability.^109^ The ε-CL-20 form crystallizes in the monoclinic space group *P*2_1_/*n* (No. 14) with four molecules per unit cell (*Z* = 4). HMX (1,3,5,7-tetranitro-1,3,5,7-tetrazocane) is highly polymorphic and exhibits multiple conformers across its four known forms.^11,110–113^ Here, we focus on δ-HMX (CSD reference code OCHTET03), which crystallizes in the hexagonal space group *P*6_1_ (No. 169) to demonstrate structure generation with six molecules per unit cell (*Z* = 6). DNI (2,4-dinitroimidazole, CSD reference code TEVHEH01) has excellent detonation properties, lower sensitivity, and higher thermal stability compared to CL-20 and HMX.^114^ It crystallizes in the orthorhombic space group *Pbca* (No. 61) with eight molecules per unit cell (*Z* = 8).^115^

The experimental structures of all six targets are successfully generated by Genarris 3.0 and retained through the steps of the clustering and down-selection workflow. In the final stage of ranking with dispersion-inclusive DFT, the experimentally observed structures of all targets are ranked as the global minimum or the second lowest-energy structure. We find that MACE-OFF23­(L) delivers variable performance for geometry relaxation and energy ranking across chemically diverse compounds. The performance for the energetic materials and Target XXII, whose chemistry is not well-represented in the training data, is worse than for aspirin and Target I. The new clustering and down-selection workflow implemented in Genarris 3.0 is able to mitigate the inconsistent performance of MACE-OFF23­(L). This makes Genarris 3.0 a versatile, robust, and efficient code for CSP^116^ and for generating molecular crystal data sets^117^ for MLIPs training.

## Methods

### Workflow Overview

Figure 2a shows an overview of the CSP workflow used in this study. Genarris 3.0 starts from a molecular structure provided by the user. Genarris 3.0 does not perform conformational sampling. For flexible molecules, Genarris 3.0 can be used with an ensemble of conformers. This has been demonstrated in the 7^th^ CSP blind test. Results for the large flexible substituted acene, Target XXVII, and Target XXXI are reported in detail in ref 89. Here, we used the molecular conformation extracted from the CSD entry, relaxed using dispersion-inclusive DFT. Genarris identifies all space groups compatible with the requested number of molecules per unit cell (*Z*) and the molecular point group symmetry, including space groups with molecules occupying special Wyckoff positions.^88^ Currently, Genarris 3.0 generates structures only with one molecule in the asymmetric unit (*Z′* = 1). A number of structures specified by the user is generated in each compatible space group.

**Figure 2:**
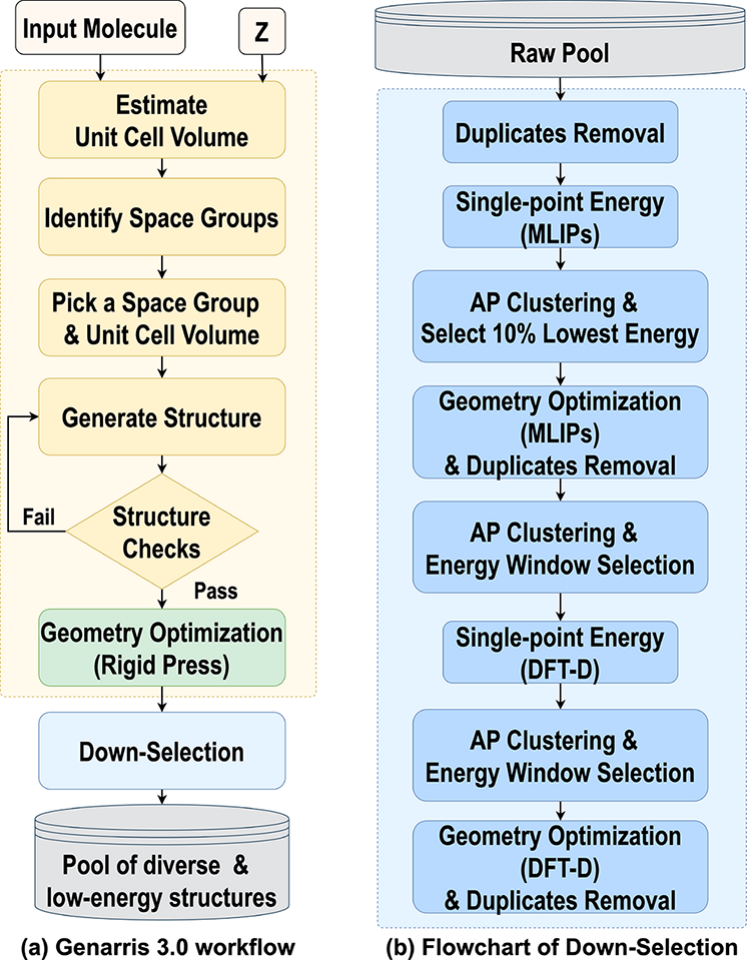
Schematic illustration of the workflow of Genarris 3.0: (a) the workflow of structure generation and (b) the down-selection workflow used here.

Structure generation starts by generating a unit cell with a volume within a normal distribution around a target value. Previously, Genarris 2.0 employed the target volume estimated by the PyMoVE machine-learned model.^94^ When using the Rigid Press algorithm (described below), the initial volume estimate is scaled by a factor of 1.5 to facilitate molecule placement. Molecules are placed in the unit cell as described in ref 88. The first molecule is randomly placed and the remaining molecules are generated based on space group symmetries. If a molecule occupies a special Wyckoff position, it is aligned with the site symmetry. The generated structure is then checked to ensure that the interatomic distance, *d*_*ij*_, between atoms *i* and *j* from different molecules is not less than *s*_r_ × (*r*_*i*_^vdW^ + *r*_*j*_^vdW^), where *r*_*i*/*j*_^vdW^ are the atomic van der Waals radii and *s*_r_ is a user-defined fraction. Here, we set *s*_r_ = 0.95 to provide sufficient distance for subsequent Rigid Press optimization. Special intermolecular distance settings are applied to strong hydrogen bonds.^88^ Structures that fail the proximity check are discarded. Structure generation continues until the requested number of structures is reached. In this work, 4000 crystal structures were generated in each compatible space group, forming the so-called “raw pool” of structures. All structures in the raw pool are initially optimized with Rigid Press. Subsequently, duplicate removal is performed within each space group by calculating the similarity via the Python Materials Genomics (Pymatgen)^118^ StructureMatcher class, using 0.5 fractional length tolerance, 0.5 site tolerance, and 10° angle tolerance. These loose tolerances are used to efficiently discard many similar configurations, significantly reducing redundancy and computational cost in subsequent screening steps.

Next, a series of user-defined screening steps can be executed using increasingly more accurate and computationally expensive methods to gradually reduce the number of structures in the pool. The down-selection workflow may be varied depending on the user’s objective. For example, a workflow intended for generating data to train MLIPs^117^ may differ from a CSP workflow.^116^ The CSP workflow used here is shown in Figure 2b. The sequence of clustering and selection steps is designed to balance considerations of structural diversity and energetic stability.

For the structures remaining after Rigid Press optimization and duplicate removal, single-point energy (SPE) calculations are performed using MACE-OFF23­(L). Afterward, affinity propagation (AP) clustering^119^ is performed with the target number of clusters set to 10% of the current structure pool. Genarris automatically adjusts the preference hyperparameter within the AP algorithm to achieve the desired number of clusters.^88^ The lowest-energy structure from each cluster is selected. The selected structures are fully relaxed with MACE-OFF23­(L), followed by an additional round of duplicate removal. Subsequently, AP clustering is performed again to produce 100 clusters. Up to 5 most stable structures within a 10 kJ/mol energy window are selected from each cluster. For the remaining structures, SPE evaluations are performed using dispersion-inclusive DFT. Then, AP clustering is performed to produce 100 clusters again, and all structures within a 10 kJ/mol energy window are selected from each cluster. We note that the number of clusters and the energy thresholds for selection in each step are a user-defined choice. Finally, the remaining structures are fully relaxed using dispersion-inclusive DFT and another round of duplicate removal is performed. This comprises the final pool of diverse and low-energy structures.

Genarris 3.0 incorporates significant code improvements. Enhanced modularity is achieved through Python’s Abstract Base Class (abc) module. This modular design simplifies the incorporation of new algorithms and optimization methods without requiring extensive modifications to the existing code. Moreover, it enables Genarris to support any MLIP model that provides a Python calculator interface for energy evaluation, thereby increasing both flexibility and usability. For example, in addition to MACE-OFF, Genarris 3.0 has been used with the AIMNet2 ^89^ and Universal Models for Atoms (UMA)^116^ MLIPs. Additionally, Genarris 3.0 features optimized multiprocessing capabilities, robust support for saving task checkpoints and restart functionality, enhanced process logging for improved monitoring and troubleshooting, and compatibility with GPU-accelerated MLIPs. These developments substantially improve the computational efficiency and performance during the structure generation and ranking tasks.

### Rigid Press

On the one hand, it may take a very large number of attempts to randomly generate close-packed molecular crystal structures while avoiding unphysical intermolecular contacts, which may lead to significant time spent on generating, checking, and discarding structures. On the other hand, increasing the target unit cell volume facilitates molecule placement, but significantly increases the time spent on relaxation of loosely packed molecular crystal structures. To address this challenge, we have developed the “Rigid Press” algorithm. Rigid Press uses a regularized hard-sphere potential to compress the unit cell based on purely geometric considerations without performing any energy evaluations. The workflow of Rigid Press is illustrated in Figure 3a. First, all the molecule pairs that are within the search radius to be considered are identified (Figure 3b). Then, an objective function formulated to minimize the unit cell volume while maintaining physical intermolecular distances is evaluated (Figure 3c). The inherently nondifferentiable hard-sphere interaction model is transformed into a smooth, differentiable function suitable for standard numerical optimization algorithms. The algorithm keeps the internal molecular geometry frozen (hence the name “Rigid Press”) as it simultaneously optimizes the molecular positions and orientations and the crystal lattice vectors to minimize the unit cell volume, while preserving the space group symmetries. Figure 3d shows representative Rigid Press optimization trajectories of aspirin Form I and ε-CL-20 (Rigid Press optimization trajectories for all other CSP targets are shown in the SI). The trajectories are characterized by a rapid initial volume reduction, indicating an effective compaction process from the initial structure (generated with an expanded volume) to the maximally close-packed final structure. The computational cost of Rigid Press optimization is lower by up to 2 orders of magnitude than relaxation using MLIPs. In addition, starting MLIP relaxation from a structure preoptimized with Rigid Press significantly reduces the number of relaxation steps required and thus the computational cost of downstream optimization, as shown in the SI. In a CSP workflow involving MLIP relaxations of thousands^116^ or even millions^89^ of putative structures this can amount to a dramatic reduction of the time to solution.

**Figure 3:**
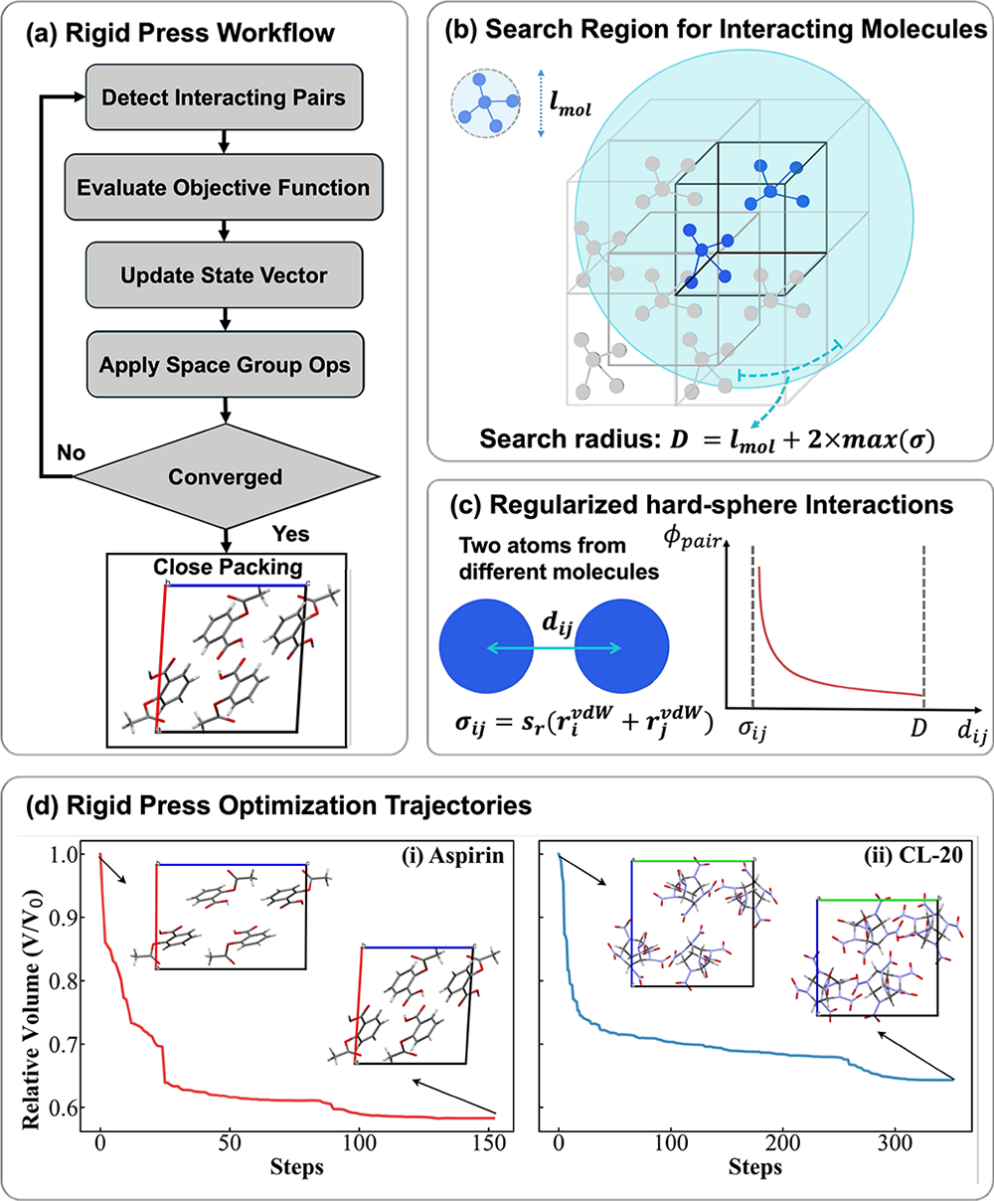
The Rigid Press algorithm: (a) overall workflow; (b) identification of interacting molecules; (c) regularized hard-sphere interaction model; and (d) representative optimization trajectories for aspirin Form I and ε-CL-20. The ratio of the unit cell volume, *V*, to the initial volume, *V*_0_, is plotted as a function of the number of optimization steps. The initial and final structures are also shown.

Within Rigid Press, a molecular crystal is represented by a state vector **s**, constructed to preserve the crystal’s space group symmetry during optimization. The state vector comprises independent lattice vectors, **L**, according to the crystal system, the position of the asymmetric unit’s center of geometry, **r**_cog_, and the orientation of the asymmetric unit represented by Euler angles, θ. The objective function, *F*(**s**), optimized by the Rigid Press algorithm is defined as

$$F(s)=V(s)+P_{\mathrm{contact}}(s)$$ 1

where *V*(**s**) denotes the unit cell volume and *P*_contact_(**s**) is the contact penalty function, which is calculated by summing over atomic penalties from all interacting molecular pairs within a specified cutoff distance:

$$P_{\mathrm{contact}}=\sum_{(A,B)\in N}\sum_{i\in A}\sum_{j\in B}\phi_{\mathrm{pair}}(d_{ij},\sigma_{ij})$$ 2

Here, the set N represents all molecular pairs that are sufficiently close for their interactions to be considered, and *d*_*ij*_ = ∥**r**_*i*_^*A*^(**s**) – **r**_*j*_^*B*^(**s**)∥ is the distance between atoms *i* and *j* belonging to different molecules *A* and *B*. σ_*ij*_ is the hard-sphere diameter for the atom pair (*i*, *j*), defined as a fraction (*s*_r_) of the sum of their van der Waals radii. Here, the default value of *s*_r_ is 0.85, with specialized *s*_r_ values applied for hydrogen bonds. These *s*_r_ values were determined by statistical analyses of experimental structures in CSD.^88^ To compute eq 2, all the molecule pairs within the interaction distance *D* need to be identified. A molecule can interact with other molecules in the unit cell, any of their periodic images or even its own periodic image. As shown in Figure 3b, the search can be limited to all the cells that are at an interacting distance *D* from the central cell. This is precomputed for a given state to reduce computational cost.

The pairwise interaction penalty ϕ_pair_(*d*, σ) is defined in a piecewise manner to ensure differentiability:

$$\phi_{pair}\left(d,\sigma \right)=\{\begin{array}{ll} \infty & \mathrm{if}\;d\leq \sigma \\ w\cdot \frac{D-d}{d-\sigma } & \mathrm{if}\;\sigma <d<D\\ 0 & \mathrm{if}\;d\geq D \end{array}$$ 3

The maximum interaction distance, *D*, is defined as *D* = *l*_mol_ + 2 × max­(σ_*ij*_), where *l*_mol_ is the diameter of the smallest sphere enclosing the molecule, defined as twice the maximum distance from the molecular center of geometry to any atom, and max­(σ_*ij*_) is the maximum interaction radius among all atom pairs. This is illustrated in Figure 3b. *w* = *k*/*N*_atoms_^2^ is a scaling factor that normalizes the contact penalty by the square of the number of atoms per molecule (*N*_atoms_) to ensure appropriate scaling, irrespective of molecular size. The constant *k* controls the relative importance of the contact penalty in the objective function in eq 1. The default value is *k* = 0.1, which was determined empirically to provide a balanced contribution from interaction penalties. Users may adjust this value as needed based on specific use cases.

The final numerical optimization employs the The Broyden-Fletcher-Goldfarb-Shanno (BFGS) algorithm^120^ implemented in the SciPy optimize class.^121^ Space group symmetries are preserved by reconstructing the full crystal coordinates from the optimized state vector, **s**, after each optimization step. Specifically, symmetry operations corresponding to the space group are applied to the optimized asymmetric unit’s position and orientation parameters, represented within the state vector **s**, to generate the complete crystal structure, thus enforcing symmetry constraints. The iterative optimization continues until reaching predefined convergence criteria, with a default tolerance of 0.01 for the gradient norm, or a maximum iteration limit of 5000. Both criteria can be customized by the user. Upon successful completion, the crystal structure is updated to reflect the optimized close-packed molecular arrangement. We have additionally implemented a faster version of the Rigid Press algorithm without symmetry constraints in C, which is interfaced with Python through Simplified Wrapper and Interface Generator (SWIG). Users may select the appropriate version based on their specific requirements.

### Computational Details

We have interfaced Genarris 3.0 with various methods for geometry optimization and energy evaluation via the Atomic Simulation Environment (ASE).^102^ All dispersion-inclusive DFT calculations were performed using the FHI-aims all-electron electronic structure code^122–124^ (version 240507). For each target, the single molecule geometry was extracted from the experimental crystal structure in CSD. The single molecule geometry was then relaxed using the PBE0 ^125^ hybrid functional, which is based on the Perdew–Burke–Ernzerhof (PBE)^126^ generalized gradient approximation, combined with the many-body dispersion (MBD) method.^127–129^ For crystal structures, single-point energy (SPE) evaluations with PBE+MBD were performed using the Tier 1 basis sets of FHI-aims and *light* numerical settings. Unit cell relaxations of the final structures using PBE+MBD were performed with the Tier 2 basis sets of FHI-aims and *tight* numerical settings. A 3 × 3 × 3 *k*-point grid was used to sample the Brillouin zone.

The MLIP employed here is the MACE-OFF^61^ pretrained transferable organic force field (OFF). MACE-OFF has three variants trained on the same SPICE 1.0 data set:^81^ small (MACE-OFF23­(S)), medium (MACE-OFF23­(M)), and large (MACE-OFF23­(L)), which differ mainly in the number of hyperparameters. Additionally, the large (*L*) variant employs an extended cutoff radius of 5 Å, utilizes more chemical channels (*k* = 192), and incorporates a higher maximum equivariant messages (max *L* = 2). These enhancements enable the MACE-OFF23­(L) model to better capture complex many-body effects and long-range interactions to achieve superior accuracy. However, this increased accuracy comes with a higher computational cost. Here, we selected MACE-OFF23­(L) because benchmark tests on the X23b data set^130^ have indicated that it provides predictions comparable in accuracy to dispersion-inclusive DFT.

All geometry relaxations with MLIPs and DFT were performed using the Broyden-Fletcher-Goldfarb-Shanno (BFGS) algorithm implemented in ASE, with a force convergence criterion of 0.01 eV/Å. We also employed the ASE FrechetCellFilter to simultaneously adjust atom positions and the unit cell, along with the ASE constraint FixSymmetry to preserve space group symmetry.

To assess the similarity between the predicted and experimentally observed crystal structures, we used the COMPACK molecular overlay method,^131^ as implemented in the Crystal Packing Similarity feature of the CSD Python API.^132^ A crystal structure is represented by a cluster of N molecules comprised of a central reference molecule and (N-1) nearest-neighbor molecules. The root mean squared deviation (RMSD) between two molecular clusters is calculated based on the molecules that match within the specified tolerances. Here, we calculated the RMSD in the atomic positions for clusters of 30 molecules, labeled as RMSD_30_. To this end, the number of matching molecules between shells of 30 molecules is extracted from the two crystal structures being compared, within 35% distance and 35° angle tolerances, excluding hydrogen atoms. This is the same comparison metric that was used in the 7^th^ CSP blind test.^36,37^

## Results and Discussion

### CSP Results

Table 1 summarizes the number of matches to the experimental structure out of the total number of generated structures in the pool at each stage of the CSP workflow. Figures 4 and 5 present the corresponding distributions of unit cell volume and space groups obtained at each stage. Similar figures for all other targets, as well as lattice parameter distributions, are provided in the SI.

**Table 1:** Summary of the number of matches to the experimental structure out of the total number of structures in the pool at each step of the Genarris 3.0 crystal structure prediction workflow for all six targets. For aspirin, matches to both polymorphs are counted.

**CSP workflow**	**aspirin**	**target XXII**	**target I**	δ**-HMX**	ε**-CL-20**	**DNI**
initial generation	0/100,004	1/104,000	0/248,000	268/52,573	0/92,000	0/232,025
Rigid Press	4/100,004	4/104,000	22/248,000	1430/52,573	6/92,000	1/232,025
duplicate removal	2/18,528	1/11,916	2/12,356	3/2,767	1/11,860	1/24,065
AP clustering @MACE-OFF23 SPE	2/1817	1/1119	1/1152	3/270	1/1219	1/2391
relaxation @MACE-OFF23 & duplicate removal	2/1567	1/1031	1/914	1/212	1/1118	1/1759
AP clustering @MACE-OFF23	2/218	1/184	1/193	1/122	1/310	1/197
AP clustering @PBE+MBD SPE	2/128	1/108	1/98	1/103	1/143	1/124
relaxation @PBE+MBD & duplicate removal	2/127	1/106	1/89	1/89	1/137	1/116

**Figure 4:**
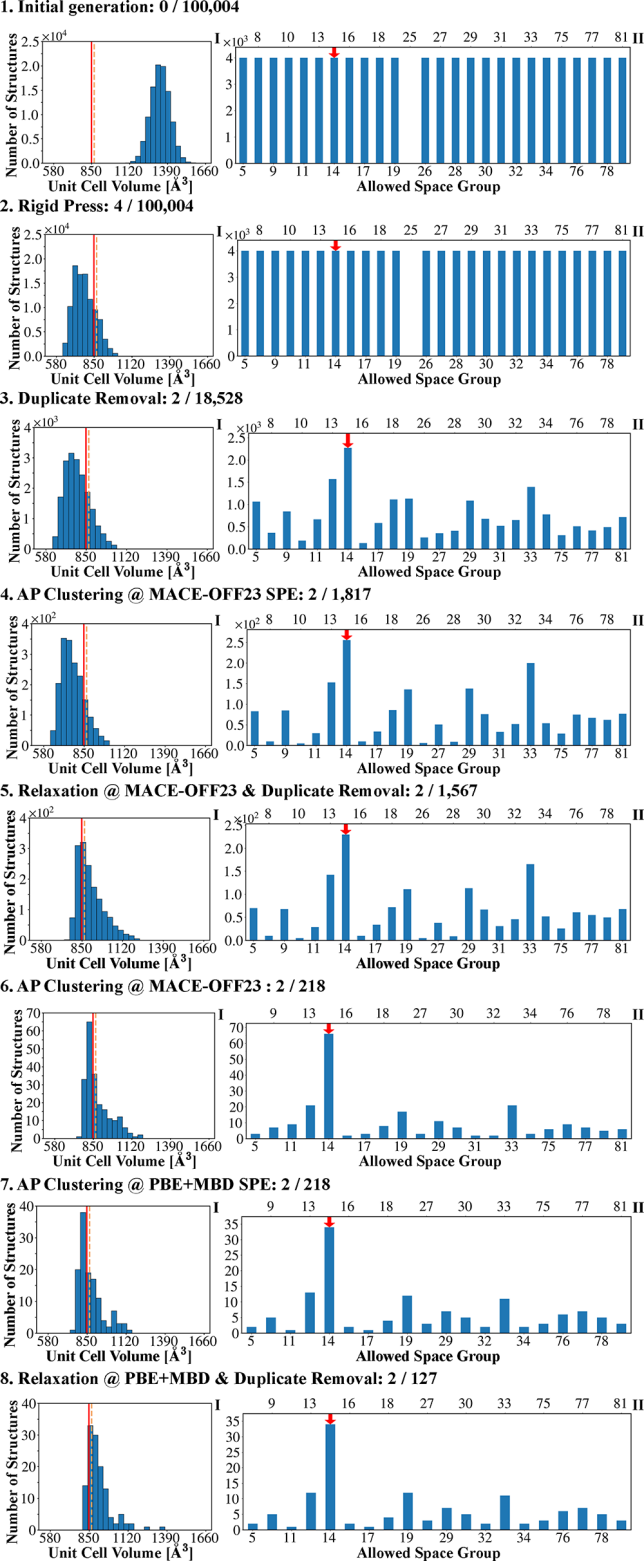
Distributions of (I) unit cell volume and (II) space groups, obtained at each step of the Genarris 3.0 workflow for aspirin with *Z* = 4. The experimental unit cell volume of Form I is indicated by a solid vertical red line and Form II is indicated by a dashed vertical orange line. The experimental space group is indicated by a red arrow.

**Figure 5:**
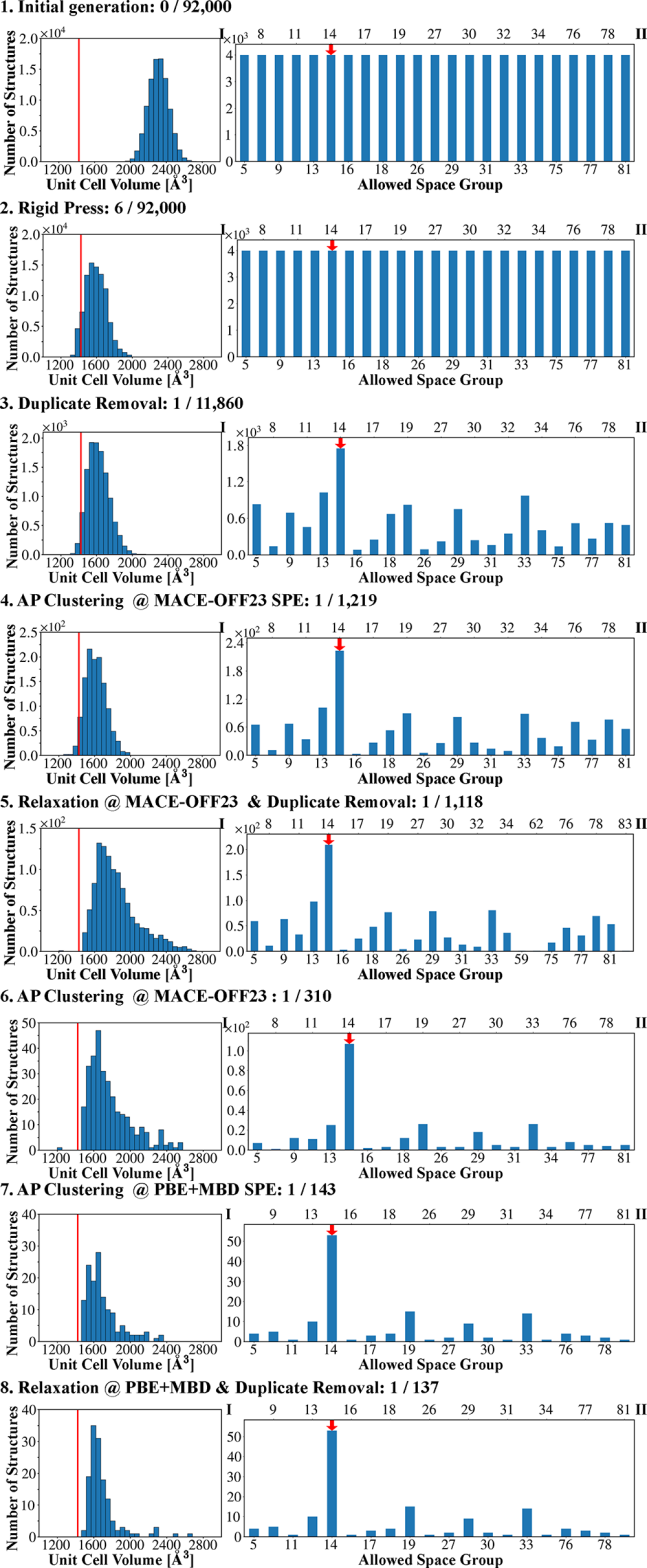
Distributions of (I) unit cell volume and (II) space groups, obtained at each step of the Genarris 3.0 workflow for ε-CL-20 with *Z* = 4. The experimental unit cell volume is indicated by a vertical red line, and the experimental space group is indicated by a red arrow.

Initially, structures are generated across all compatible space groups for each target, as indicated by the uniform space group distributions in Figures 4 and 5. For example, aspirin structures with *Z* = 4 were generated across 26 compatible space groups and DNI structures with *Z* = 8 were generated across 63 compatible space groups (see SI). Because the initial generation is performed with an increased target volume, the unit cell volume histograms are significantly overestimated compared to the experimental values at this stage. For most targets, no matches are found after initial generation. For Target XXII, one match is found out of 104,000 structures. For δ-HMX, 268 matches are found out of 52,573 generated structures. This higher match rate is likely because the highly constrained space group symmetry (*P*6_1_, No. 169) limits the degrees of freedom for the molecular positions and orientation, which reduces the configuration space to be searched and increases the likelihood of generating the correct structure.

After optimization with Rigid Press, the unit cell volume histograms in Figures 4 and 5 are closer to the experimental values (see also SI). It is interesting to note that for aspirin, Target XXII, and Target I the unit cell volumes are somewhat underestimated after Rigid Press, whereas for the very dense energetic materials the unit cell volumes after Rigid Press are very close to the experimental values for δ-HMX and DNI and still slightly overestimated for ε-CL-20. The space group histograms are unchanged because Rigid Press preserves the space group symmetry. Importantly, after Rigid Press, matches are found for all targets. For most targets, only a handful of matches are found out of ∼10^5^ generated structures. For Target I, 22 matches are found out of 248,000 structures. The higher number of matches may be attributed to the molecule’s rigidity and the common *Pbca* (No. 61) space group, which facilitates good packing. For δ-HMX, the number of matches increases to 1430. There is a clear distinction between structures that are difficult to generate, and are generated very rarely, such as DNI with a single match out of 232,025 structures, compared to structures that are easy to generate and are generated frequently, such as δ-HMX. A comparison for ε-CL-20 between workflows started from the same initial pool with and without Rigid Press is provided in the SI, demonstrating that a match to the experimental structure can only be found with Rigid Press.

At this point, duplicate removal drastically reduces the number of structures in the pool without significantly changing the volume distributions. For Target XXII, ε-CL-20 and DNI, the reduction is by a factor of 8–10. For aspirin, the reduction is by a smaller factor of 5. The greatest reductions are for Target I and δ-HMX by a factor of 19–20. We consider a large number of duplicates as an indication that the configuration space is exhaustively sampled. To reduce the number of duplicates, the user can reduce the number of structures generated in each space group. After this step, the space group distributions are no longer uniform because more duplicates are generated in some space groups than in others. Certain space groups, such as *P*2/*m* (No. 10) and *P*222 (No. 16), include more special Wyckoff sites, limiting the number of available general positions. As a result, when Genarris attempts to place molecules on the general Wyckoff position of these space groups fewer unique arrangements are possible. Additionally, tetragonal and orthorhombic crystal systems with higher-symmetry space groups (e.g., Nos. 75–81) impose stricter symmetry constraints, leading to fewer unique crystal packing arrangements. Symmetry elements such as mirror planes and inversion centers greatly increase the multiplicity of equivalent positions and thereby increase the number of duplicates generated. For instance, space group *Cm* (No. 8) is *C*-centered, causing each initial placement to generate multiple symmetry-equivalent structures. In all cases, a large number of structures are retained in the space group of the experimental structure(s). After duplicate removal only one match to the experimental structure remains for Target XXII, ε-CL-20, and DNI. For aspirin, one match remains for each polymorph. For Target I and δ-HMX, two and three matches are left, respectively.

After AP clustering and selection based on MACE-OFF23­(L) single-point energies, the number of structures is reduced to 10% while retaining the matches to experiment for all targets. After full unit cell relaxation with MACE-OFF23­(L), the unit cell volume histograms in Figures 4 and 5 shift to higher values. Duplicate removal further reduces the number of structures only slightly. This is an indication that the structures remaining after the first clustering and selection step are already unique and structurally diverse. In the two subsequent clustering and selection steps, the number of remaining structures varies between targets, depending on the number of structures within the 10 kJ/mol energy window (see further discussion below). With each clustering and down-selection step, the volume distributions become narrower, while retaining a large number of structures in the experimental space groups.

Figure 6 shows the final potential energy landscapes obtained with PBE+MBD for all six targets. For all targets, except for Target XXII, the experimentally observed forms are ranked as the global minimum. For Target XXII, it has been shown previously that the experimental structure is ranked as the global minimum only with PBE0+MBD.^133^ For aspirin, Form II is predicted to be more stable than Form I by 0.69 kJ/mol. Experimental observations suggest that Form I is more stable than Form II at 300 K.^134–136^ Previous computational studies using dispersion-corrected DFT^134,137^ and fragment-based hybrid quantum classical methods^138,139^ have reported that the two polymorphs are very close in energy. These studies have also shown that the relative stability of Form I and Form II depends on the choice of method and whether free energy corrections are applied. Figure S6 in the Supporting Information shows that PBE+MBD free energy at 300 K, calculated using the quasi-harmonic approximation (QHA), as described in ref 54., predicts Form I to be more stable than Form II by 1.10 kJ/mol. For the energetic materials CL-20 and DNI, putative low-energy, high-density structures are found, with lattice energies2.69 kJ/mol above the predicted ε form of CL-20 and 1.69 kJ/mol above the experimentally observed form of DNI.

**Figure 6:**
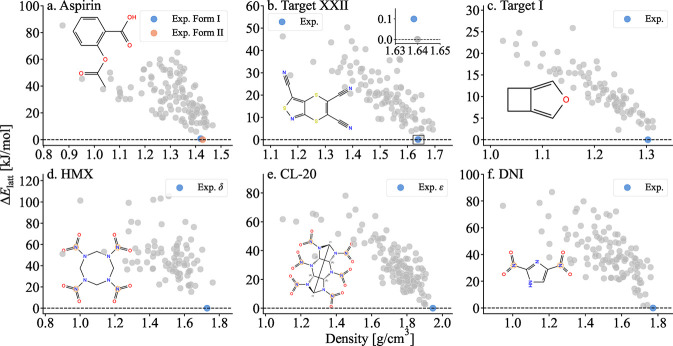
Energy as a function of density, calculated at the PBE+MBD level of theory for the six benchmark targets: (a) aspirin, (b) Target XXII, (c) Target I, (d) HMX, (e) CL-20, and (f) DNI. Experimentally observed polymorphs are highlighted in color, and putative crystal structures are shown in gray. The molecular structures are also shown.

### MACE-OFF Performance

In the following, we assess the performance of MACE-OFF23­(L) for geometry relaxation and energy ranking by comparing the results with PBE+MBD, which we treat as the ground truth for putative crystal structures. Figure 7 shows the distributions of RMSD_30_ values obtained by comparing the structures relaxed with MACE-OFF23­(L) to the structures relaxed with PBE+MBD, starting from the same initial configuration. We consider structures as matching if 30 molecules are overlaid and RMSD_30_ < 1 Å, indicating that both methods produce similar relaxed configurations. Only structures that match their DFT-relaxed counterparts based on the RMSD_30_ values are included in our analysis. We use this as a metric for assessing how closely MACE-OFF23­(L) reproduces the PBE+MBD potential energy surface (PES). If the PBE+MBD PES is reproduced well, then we expect MACE-OFF23­(L) to arrive at the same local minimum structure. We note that in the ranking stage of the 7^th^ CSP blind test, it was considered a failure if some of the relaxed structures obtained with a certain method no longer matched the initial structures provided by the CCDC.^37^ In particular, with some of the MLIPs used therein, the experimental structures of some of the targets could no longer be matched. In contrast, relaxation failures did not occur with any of the dispersion-inclusive DFT methods used therein (nor with the AIMNet2 MLIPs).

**Figure 7:**
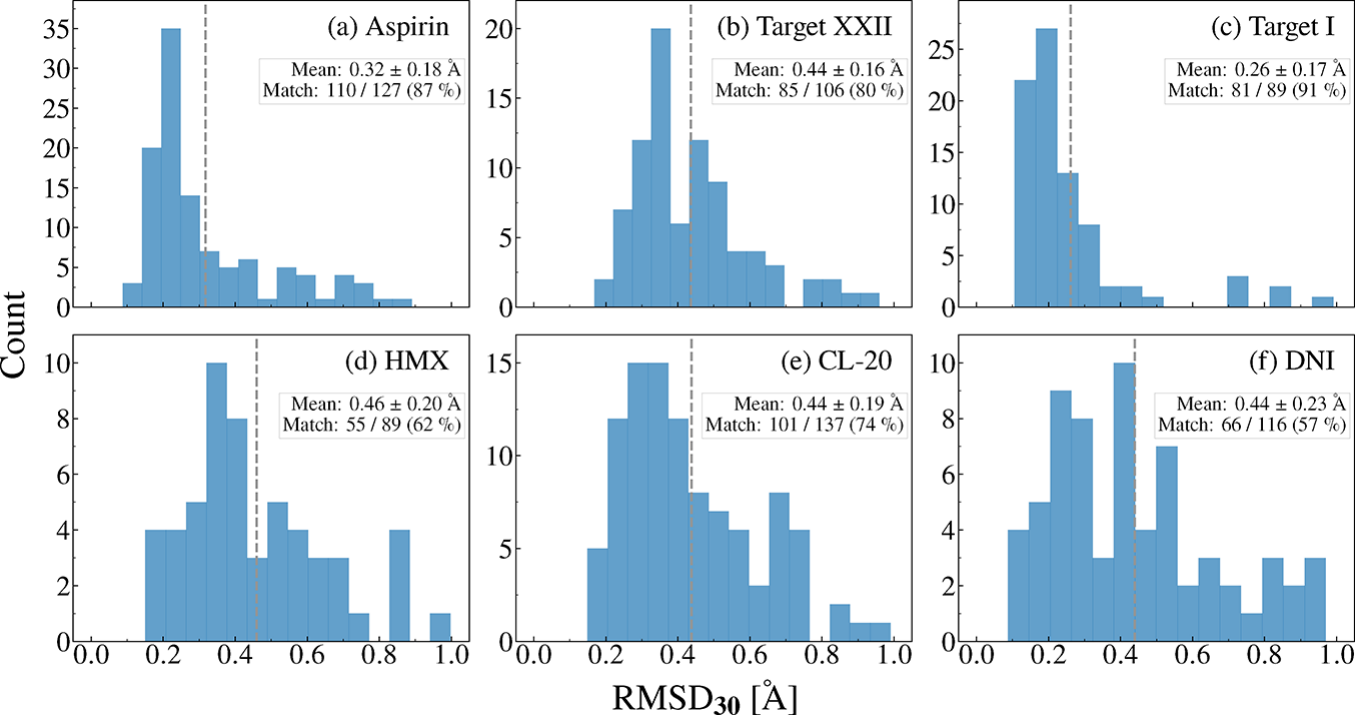
RMSD_30_ histograms of the relaxed crystal structures obtained with the MACE-OFF23­(L) model compared to those obtained with PBE+MBD, starting from the same initial configuration, for (a) aspirin, (b) Target XXII, (c) Target I, (d) δ-HMX, (e) ε-CL-20, and (f) DNI. The mean RMSD_30_ is indicated by a vertical dashed line and the match rate is also shown.

There is significant variation in the relaxation performance of MACE-OFF23­(L) across targets. For aspirin and Target I, the structures relaxed with MACE-OFF23­(L) are largely in excellent agreement with PBE+MBD. The RMSD_30_ histograms peak around 0.2 Å and most structures have an RMSD_30_ below 0.3 Å. For aspirin, 17 mismatches occurred out of 127 structures, corresponding to 86.6% match rate. For Target I, there were 8 mismatches out of 89 structures, resulting in a 91.0% match rate. For Target XXII, the relaxation performance of MACE-OFF23­(L) is somewhat worse. Its RMSD_30_ histogram peaks around 0.35 Å, with the majority of structures possessing RMSD_30_ values below 0.6 Å. Target XXII also has a somewhat lower match rate than aspirin and Target I, with 21 mismatches out of 106 structures amounting to 80.2%.

For the three energetic materials, the relaxation performance of MACE-OFF23­(L) is markedly worse. For δ-HMX, ε-CL-20, and DNI, the RMSD_30_ distributions are broader, peak around 0.3–0.4 Å, and a significant number of structures have RMSD_30_ values above 0.6 Å. The worse relaxation performance also manifests in a significantly lower match rates for these targets. For δ-HMX there were 34 mismatches out of 89 structures (61.8%), for ε-CL-20 there were 36 mismatches out of 137 structures (73.7%), and for DNI there were 50 mismatches out of 116 structures (56.9%). Across all targets, we observe a weak correlation between the relaxation performance and the relative lattice energies, where more stable structures tend to have lower RMSD_30_ values, as shown in the SI.

In Figure 8, the performance of MACE-OFF23­(L) in stability ranking is assessed by comparing the relative lattice energies of structures relaxed with MACE-OFF23­(L) against those relaxed with PBE+MBD, which serve as the reference. For aspirin and Target I, MACE-OFF23­(L) performs well. The MAE and RMSE values are below 5 kJ/mol and the Kendall ranking correlation score is above 0.7. It is also apparent in Figure 8a,c that the data points are concentrated quite close to the parity line. For both of these targets, MACE-OFF23­(L) ranks the experimentally observed structures as the lowest in energy. For Target XXII, ε-CL-20, and DNI the performance of MACE-OFF23­(L) is significantly worse, with MAE and RMSE values ranging between 9 and 13 kJ/mol and Kendall ranking correlation scores of 0.5–0.6. It is also evident in Figure 8b,e,f that the data points are scattered farther away from the parity line compared to aspirin and Target I. The worst performance is found for δ-HMX with MAE and RMSE values above 20 kJ/mol and a Kendall ranking correlation score below 0.4. For Target XXII, the experimental structure is ranked as #2 in agreement with PBE+MBD. For the three energetic materials the experimental structures are ranked quite poorly by MACE-OFF23­(L), as #12 for δ-HMX, #8 for ε-CL-20, and #19 for DNI. Similar performance trends are also evident from the comparison of the relative energy vs density landscapes obtained with MACE-OFF23­(L) to PBE+MBD, shown in the SI.

**Figure 8:**
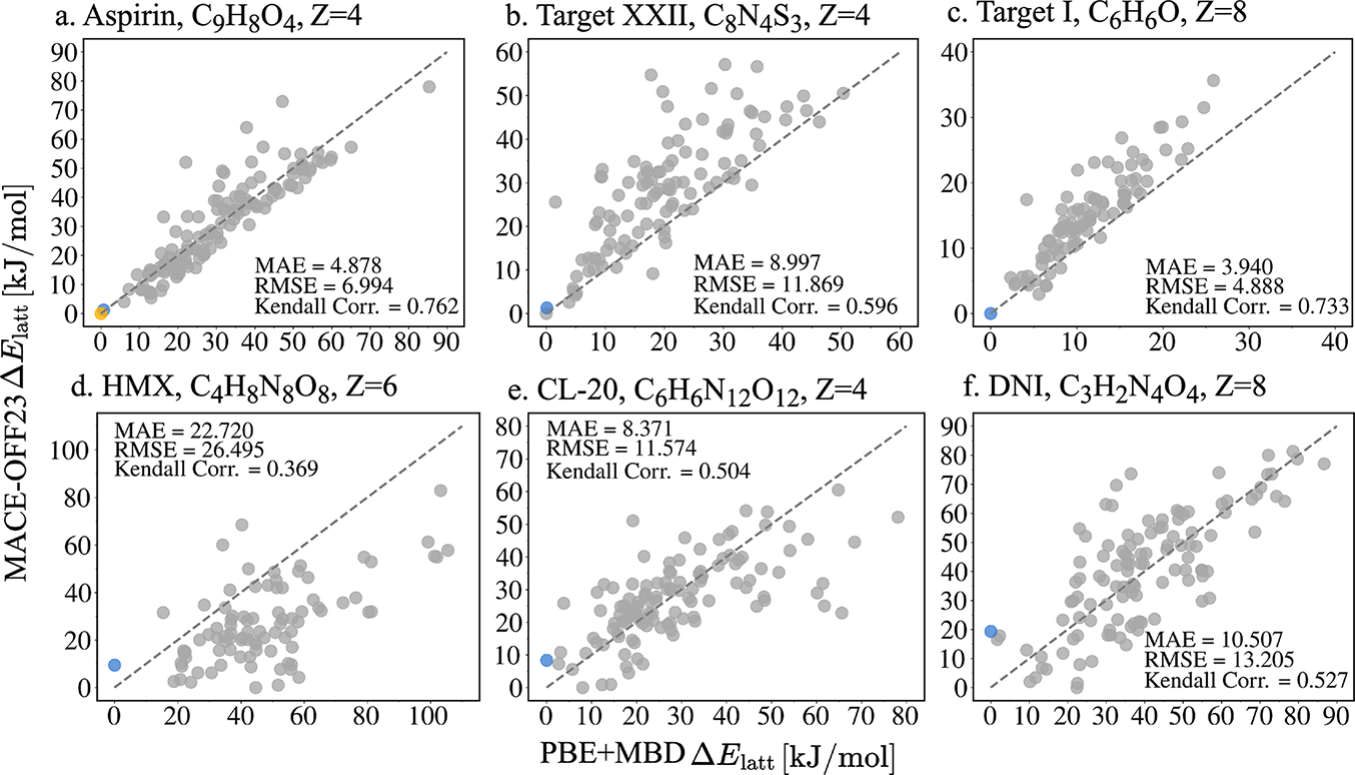
Relative lattice energies Δ*E*_latt_ obtained with the MACE-OFF23­(L) model, compared to those calculated using PBE+MBD for (a) aspirin, (b) Target XXII, (c) Target I, (d) δ-HMX, (e) ε-CL-20, and (f) DNI. The experimentally observed structures are indicated in color. The mean absolute error (MAE), root mean squared error (RMSE), and Kendall correlation score are also shown.

The variable performance of MACE-OFF23­(L) can be attributed to the similarities and differences between our target molecules and the compounds contained in the SPICE training data set. The SPICE 1.0 data set mainly comprises drug-like molecules. This explains the good performance of the MACE-OFF23­(L) model for the pharmaceutical target, aspirin, and Target I. The chemistry of Target XXII and, to a greater extent, the energetic molecules is very different from typical pharmaceutical compounds. Energetic materials, which feature a high concentration of nitrogen-containing groups, are underrepresented in the SPICE data set. Our findings are in agreement with a recent study,^140^ which also reported poor performance of MACE-OFF23­(L) for molecules whose chemistry differs from the SPICE data set, including Target XXII. These results highlight the limitations in the transferability of the MACE-OFF MLIPs across chemically diverse compounds.

If the out-of-the-box performance of a general-purpose MLIP is inadequate for a compound of interest, it is possible to train a system-specific AIMNet2 model.^89^ To demonstrate this, a system-specific AIMNet2 potential was trained for CL-20, as described in the SI. Figure S4 shows that the system-specific AIMNet2 potential delivers better relaxation performance than MACE-OFF23­(L), as evidenced by the higher match rate and lower RMSD_30_ with respect to PBE+MBD. Figure S5 shows that AIMNet2 provides better performance than MACE-OFF23­(L) for relative energy ranking, as indicated by a lower relative energy MAE and a higher Kendall ranking correlation with respect to PBE+MBD. Notably, AIMNet2 significantly improves the relative energy and ranking of the experimental structure, which is ranked as #4 with a relative energy of 2.50 kJ/mol above the global minimum. The system-specific AIMNet2 model could be improved even further with additional training using active learning to select the most informative configurations.^89^

The clustering and down-selection workflow used here can mitigate to some extent the limitations of MACE-OFF23­(L), as illustrated in Figure 9 for DNI. Figure 9a shows the landscape of relative energy as a function of density obtained after relaxation with MACE-OFF23­(L) and duplicate removal (step 5 in Figure S12). The best match to the experimental structure (colored in red) is ranked as #79, 19.3 kJ/mol above the MACE-OFF23­(L) lowest-energy structure. Single-point energy calculations using PBE+MBD on the MACE-OFF23­(L) relaxed structures reveal significant changes in the relative energy ranking, as shown in Figure 9b. The experimental structure becomes the global minimum and the MACE-OFF23­(L) lowest-energy structure (colored in blue) is 21.13 kJ/mol higher in energy. This highlights that inaccuracies in the MACE-OFF23­(L) energy ranking could potentially lead to the loss of important structures in the early stages of hierarchical CSP workflows that employ energy cutoffs to pass structures from one stage to the next. Here, the experimental structure is retained thanks to our clustering and down-selection approach. Figure 9c shows the MACE-OFF23­(L) relative energy as a function of density for the cluster that contains the experimental structure after the AP clustering step. The experimental structure is ranked second in its cluster, after the structure colored in green. Because our procedure is to select up to 5 structures within a 10 kJ/mol window, rather than selecting only the most stable structure out of each cluster, the experimental structure is retained, despite the limitations of MACE-OFF23­(L). This selection method enhances the robustness of our down-selection workflow. Similar analysis for all other targets is provided in the SI, showing that the clustering and down-selection procedure is particularly beneficial for the other two energetic targets δ-HMX and ε-CL-20, whose experimental structures are poorly ranked by MACE-OFF23­(L).

**Figure 9:**
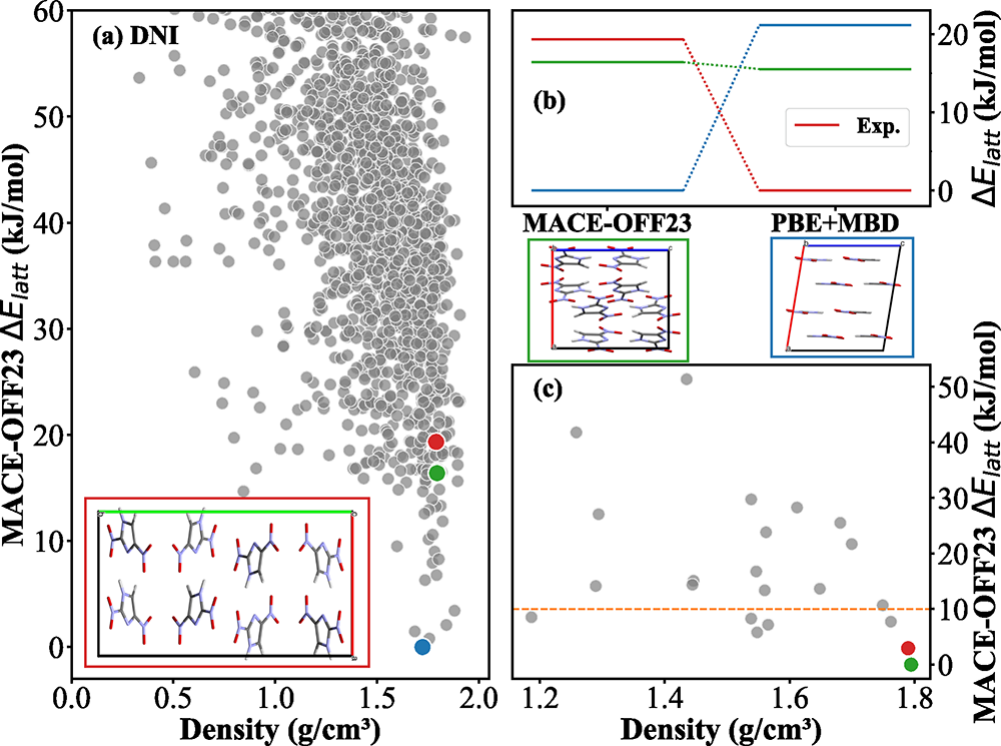
Clustering and down-selection workflow for DNI: (a) relative lattice energies computed using MACE-OFF23­(L) as a function of crystal density after relaxation with MACE-OFF23­(L). The experimental structure (red), the MACE-OFF23­(L) lowest-energy structure (blue), and the lowest-energy structure in the cluster containing the experimental structure (green) are highlighted. (b) Comparison of relative lattice energies computed with MACE-OFF23­(L) and PBE+MBD for the three structures, which are also shown. The PBE+MBD calculations were performed on the structures relaxed with MACE-OFF23­(L). (c) MACE-OFF23­(L) relative energy as a function of crystal density for the cluster containing the experimental structure. The orange dashed line indicates the 10 kJ/mol energy threshold.

## Conclusion

In summary, we have presented a new version of our open-source molecular crystal structure generator, Genarris 3.0. In this version, we have implemented the Rigid Press algorithm, which efficiently generates close-packed molecular crystal structures by using a regularized hard-sphere potential to compress the unit cell, while preserving the space group symmetries. In addition, we have interfaced Genarris 3.0 through ASE with a variety of methods for geometry relaxation and energy evaluation, including DFT and MLIPs, offering the user maximal flexibility. We have introduced a new CSP workflow of clustering and down-selection to gradually reduce the number of structures evaluated with increasingly accurate and more computationally expensive methods. For demonstration purposes, we employed the MACE-OFF general-purpose MLIPs in the early stages of the workflow.

Genarris 3.0 successfully generated the experimentally observed crystal structures of the pharmaceutical aspirin, the two past blind test targets, Target I and Target XXII, and the three energetic materials δ-HMX, ε-CL-20, and DNI. The best matched structures were retained throughout the clustering and down-selection workflow. MACE-OFF23­(L) delivered variable performance for relaxation and energy ranking across chemically diverse compounds. The performance for Target XXII and the energetic materials, whose chemistry is not well-represented in the SPICE 1.0 data set, was worse than for aspirin and Target I. This has highlighted some limitations in the transferability of general-purpose MLIPs. We have demonstrated that our clustering and down-selection workflow was able to mitigate the inaccuracy of MACE-OFF23­(L), especially for the energetic materials, whose experimental structures were significantly misranked.

Our results emphasize that although general-purpose MLIPs, such as MACE-OFF, can considerably accelerate early stage CSP workflows, dispersion-inclusive DFT remains indispensable for accurate final ranking. Based on our findings, we suggest exercising caution when using general-purpose MLIPs for CSP. We recommend careful validation of the performance of general-purpose MLIPs on a case-by-case basis, especially if the chemistry of the materials of interest is significantly different than the materials represented in the training data. If their out-of-the-box performance is inadequate for the materials of interest, alternative solutions, such as system-specific AIMNet2 potentials^89^ may be considered.

In conclusion, Genarris 3.0 is a versatile and robust open-source code for molecular crystal structure generation. Genarris 3.0 is able to generate structures in all space groups, including with structures occupying special Wyckoff positions. It offers the user maximal flexibility in the choice of method for relaxations and energy evaluations and in the design of CSP workflows. For flexible molecules, Genarris 3.0 may be started with an ensemble of conformers, as we had previously demonstrated within the 7^th^ CSP blind test.^89^ Future improvements include generating structures with more than one molecule in the asymmetric unit. Genarris 3.0 may be used to perform CSP by random sampling,^116^ to generate initial structure pools for other CSP methods,^141^ and to generate data sets for MLIP training.^117^

## Supplementary Material



## Data Availability

Genarris 3.0 is available on GitHub (https://github.com/Yi5817/Genarris) and through the Web site (https://www.noamarom.com/software/genarris/) under the BSD-3-Clause license. The putative structures relaxed with MACE-OFF23­(L) and PBE+MBD for the 6 target molecules, and system-specific AIMNet2 model for ε-CL-20 in this study are available at (https://github.com/Yi5817/Genarris).

## Notes

Additional analysis of MACE-OFF optimization with and without Rigid Press; Rigid Press optimization trajectories; comparison of Genarris 3.0 CSP workflows with and without Rigid Press by two MLIPs; additional details on on density and relative lattice energy comparison for different workflows and MLIPs; comparison of AIMNet2 and PBE+MBD; additional details on unit cell volumes, lattice parameters, and space groups distributions at each step of the CSP workflow in Genarris 3.0; ranking of low-energy structures with different dispersion-inclusive DFT methods and thermal corrections; tabulated lattice parameters and RMSD_30_ for polymorphs obtained by MACE-OFF23­(L) and PBE+MBD; correlation analysis between RMSD_30_ values and relative lattice energies; density and relative lattice energy comparison between MACE-OFF23­(L) and PBE+MBD; and additional analysis of the clustering and down-selection workflow (PDF)

## #

Department of Physics and Astronomy, Texas A&M University, College Station, Texas 77843, United States
