# Supplementary material for: Genarris 3.0: Generating Close-Packed Molecular Crystal Structures with Rigid Press
Source: J Chem Theory Comput. 2025 Oct 30;21(21):11318–32. doi: 10.1021/acs.jctc.5c01080 (PMC12613321; doi:10.1021/acs.jctc.5c01080)
Supplement: Supplementary file 1 [file ct5c01080_si_001.pdf]

# Supplementary Information

# Genarris 3.0: Generating Close-Packed Molecular Crystal Structures with Rigid Press

Yi Yang,[†] Rithwik Tom,[‡] Jose A. G. L. Wui,[¶,⊥] Jonathan E. Moussa,[§] and Noa Marom[*,‡,†,‖]

†*Department of Materials Science and Engineering, Carnegie Mellon University, Pittsburgh, PA 15213, USA*

‡*Department of Physics, Carnegie Mellon University, Pittsburgh, PA 15213, USA*

¶*Department of Physics, University of Texas at Austin, Austin, TX 78712, USA*

§*Molecular Sciences Software Institute, Blacksburg, VA 24060, USA*

‖*Department of Chemistry, Carnegie Mellon University, Pittsburgh, PA 15213, USA*

⊥*Current address: Department of Physics and Astronomy, Texas A&M University, College Station, TX 77843, USA*

E-mail: nmarom@andrew.cmu.edu

# Rigid Press

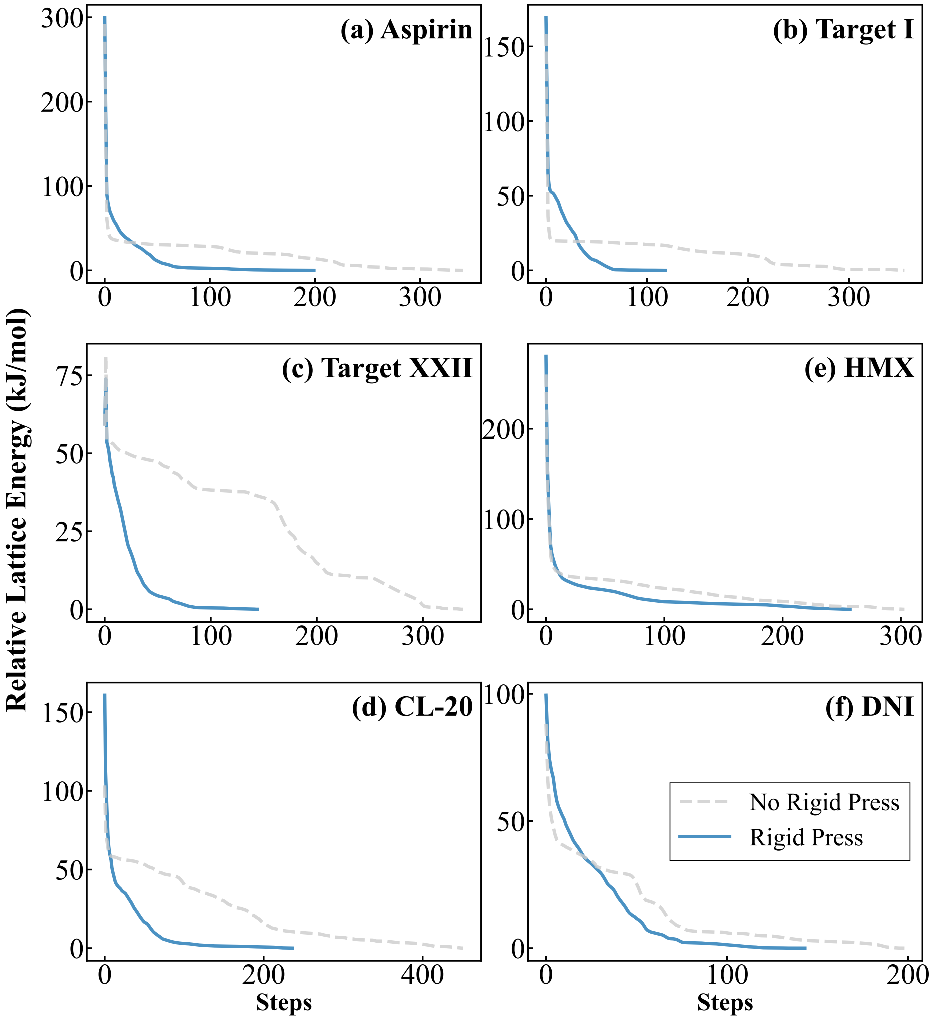


Figure S1: MACE-OFF optimization trajectories for (a) Aspirin, (b) Target I, (c) Target XXII, (d) $\varepsilon$-CL-20, (e) $\delta$-HMX, (f) DNI. The dashed gray lines represent trajectories started from initial structures without Rigid Press optimization and the solid blue lines correspond to trajectories started from structures optimized with Rigid Press. Pre-optimization of generated structures with Rigid Press significantly reduces the number of relaxation steps and thus the computational cost.

Table S1: Relaxation performance of MACE-OFF with the BFGS optimizer when starting from initial structures with and without Rigid Press pre-optimization on an NVIDIA GH200 GPU. The check marks indicates whether the final relaxed structure was a match to the experimental structure.

| Target | Rigid Press | Time (s) | Steps | ΔTime | ΔSteps |
|---|---|---|---|---|---|
| Aspirin | - | 177.64 | 341 | - | - |
| | ✓ | 108.58 | 199 | -38.9% | -41.6% |
| Target I | - | 222.01 | 355 | - | - |
| | ✓ | 80.72 | 118 | -63.6% | -66.8% |
| Target XXII | - | 114.59 | 338 | - | - |
| | ✓ | 49.63 | 144 | -56.7% | -57.4% |
| $\delta$-HMX | - | 305.07 | 303 | - | - |
| | ✓ | 262.78 | 257 | -13.9% | -15.2% |
| $\varepsilon$-CL20 | - | 383.36 | 451 | - | - |
| | ✓ | 210.92 | 236 | -45.0% | -47.7% |
| DNI | - | 120.88 | 198 | - | - |
| | ✓ | 89.62 | 143 | -25.9% | -27.8% |

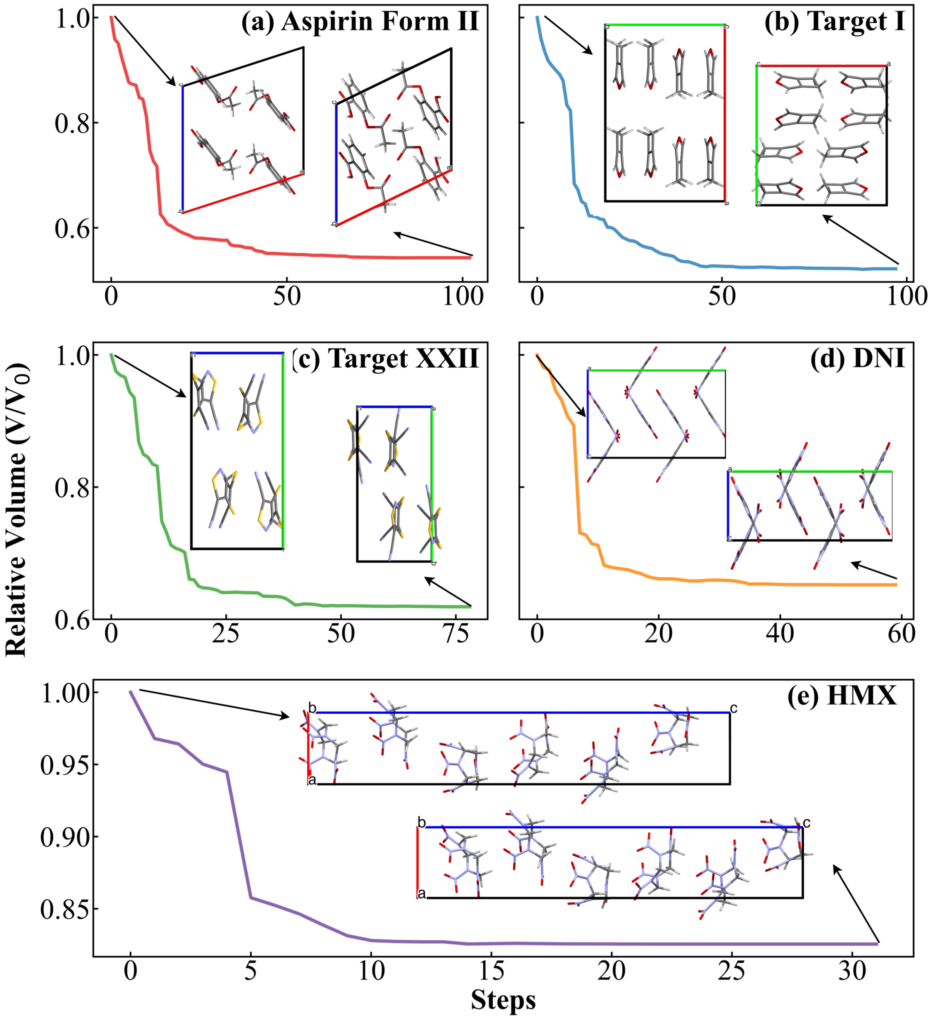


Figure S2: Rigid Press optimization trajectories for (a) Aspirin Form II, (b) Target I, (c) Target XXII, (d) DNI, (e) $\delta$-HMX. The initial and final structures are also shown.

# Workflow

To demonstrate the importance of Rigid Press, a similar workflow was run for $\varepsilon$-CL20 without the Rigid Press step. The workflow was started from the same initial pool of generated structures and proceeded directly to duplicate removal and all the subsequent steps. The results are summarized in Table S2 and Figure S3. Without Rigid Press a match to the experimental structure is not found after relaxation with MACE-OFF23, nor after final relaxation with PBE+MBD. This is because relaxation with MACE-OFF23 from the initial generated structure without pre-optimization with Rigid Press does not converge to the experimental structure, as shown in Table S1. Moreover, including the Rigid Press step reduces the time spent on relaxations with MACE-OFF23 from 24 s per structure to 20 s per structure, on average. We attribute this to a reduction in the number of relaxation steps needed, as shown in Figure S1 and Table S1.

Table S2: Comparison of Genarris 3.0 CSP workflows with and without Rigid Press (RP), evaluated using two machine-learning interatomic potentials (MLIPs): MACE-OFF23 and system-specific AIMNet2 for $\varepsilon$-CL20. Reported values include the number of matched structures, relaxation times (h), and the final $\text{RMSD}_{30}$ at the MLIP stage.

| **Genarris 3.0 Workflow** | **With RP @MACE-OFF23** | | **No RP @MACE-OFF23** | | **With RP @AIMNet2** | |
|---|---|---|---|---|---|---|
| | **#** | **Time (h)** | **#** | **Time (h)** | **#** | **Time (h)** |
| Initial generation | 0/92,000 | - | 0/92,000 | - | 0/92,000 | - |
| Rigid Press | 6/92,000 | - | - | - | 6/92,000 | - |
| Duplicate removal | 1/11,860 | - | 0/16,413 | - | 1/11,860 | - |
| AP clustering @MLIPs SPE | 1/1,219 | - | 0/1,613 | - | 1/1,100 | - |
| Relaxation @MLIPs & duplicate removal | 1/1,118 | 6.77 | 0/1,489 | 10.57 | 1/962 | 6.14 |
| AP clustering @MLIPs | 1/310 | - | 0/379 | - | 1/157 | - |
| AP clustering @PBE+MBD SPE | 1/143 | - | 0/131 | - | - | - |
| Relaxation @PBE+MBD & duplicate removal | 1/137 | - | 0/129 | - | 1/149 | - |
| **$\text{RMSD}_{30}$@MLIPs (Å)** | 0.286 | | - | | 0.261 | |

To demonstrate the effect of the choice of MLIP, a system-specific AIMNet2 potential was trained for CL-20. System-specific AIMNet2 model was trained on molecular clusters ($n$-mers) extracted from crystal structures generated by Genarris. Each $n$-mer contained the reference molecule unit and $n - 1$ of its neighbors. Additional $n$-mers were sampled from short molecular dynamics trajectories using the GFNFF-xTB force field[1] to capture out-of-equilibrium conformations. After sampling, single-point calculations were performed to obtain the energy, atomic forces, partial charges, and dipole data. To expedite training, we first calculated at the GFN2-xTB[2] level to obtain a low-fidelity model, which provides faster convergence and extensive coverage of the potential energy surface (PES). Here, we used tight-binding framework (TBLITE), which is a Python implementation of GFN methods, to perform molecular dynamics and force field calculations. We then applied transfer learning to refine this model using a subset of training data calculated at the PBE level with the Karlsruhe triple-zeta basis set and two sets of polarization functions (def2-TZVPP)[3,4] using the Orca 6.0.0 software.[5,6] The final model includes Becke-Johnson-damped D3 (D3BJ)[7] dispersion correction to account for long-range van der Waals interactions. The ystem-specific AIMNet2 model for $\varepsilon$-CL20 was trained on a total of $3.5 \times 10^5$ $n$-mers, comprising $5 \times 10^4$ monomers, $1 \times 10^5$ dimers, $1 \times 10^5$ trimers, and $1 \times 10^5$ tetramers. Of these, only 10 % were calculated at the DFT level.

The workflow was run starting from the same initial pool, using system specific AIMNet2 instead of MACE-OFF23, starting from the fourth step of AP clustering based on MLIP SPE. The results are summarized in Table S2. The choice of MLIP affected the results of clustering and selection, producing a somewhat different final population, as shown in Figure S3. In both cases, the match to the experimental structure was found after MLIP relaxation and retained through the subsequent steps. The AIMNet2 model is faster in practice because, unlike equivariant architecture models, it avoids costly high-order tensor operations. In addition, the model is more compact than MACE-OFF23(L), with 2,205,768 parameters compared to 4,707,312, leading to a slight speedup of the relaxation.

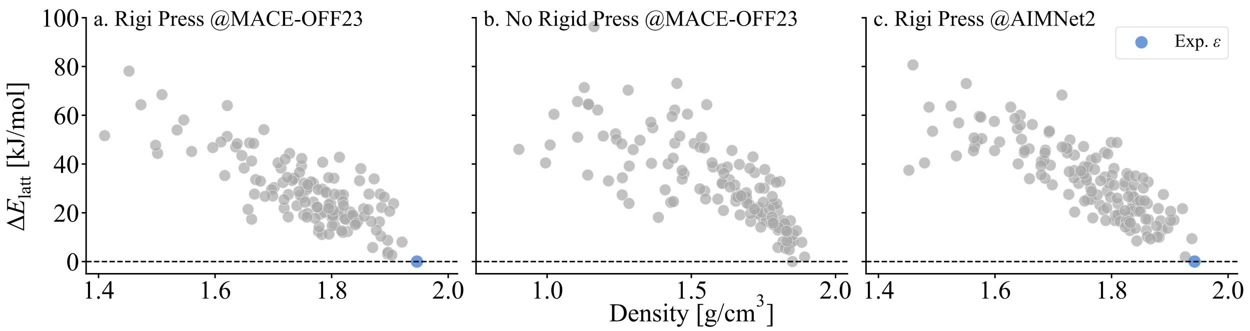


Figure S3: Energy landscapes of $\varepsilon$-CL20, calculated at the PBE+MBD level of theory for CSP workflows with (a) Rigid Press using MACE-OFF23, (b) no Rigid Press using MACE-OFF23, and (c) Rigid Press using AIMNet2. The experimental structure is indicated by the blue marker.

Figure S4 compares the relaxation performance of the system-specific AIMNet2 potential to MACE-OFF23(L) for $\varepsilon$-CL20. The AIMNet2 potential delivers better relaxation performance, as evidenced by the higher match rate and lower $\text{RMSD}_{30}$ with respect to PBE+MBD. Figure S5 compares the energy ranking performance of the system-specific AIMNet2 potential to MACE-OFF23(L) for $\varepsilon$-CL20. AIMNet2 produces a lower relative energy MAE and a higher Kendall ranking correlation than MACE-OFF23(L), with respect to PBE+MBD. Notably, AIMNet2 significantly improves the relative energy and ranking of the experimental structure, which is ranked as # 4 with a relative energy of 2.50 kJ/mol above the global minimum, compared to #8 about 10 kJ/mol above the global minimum with MACE-OFF23(L). The system-specific AIMNet models could be further improved by additional training using active learning to select the most informative configurations.[8]

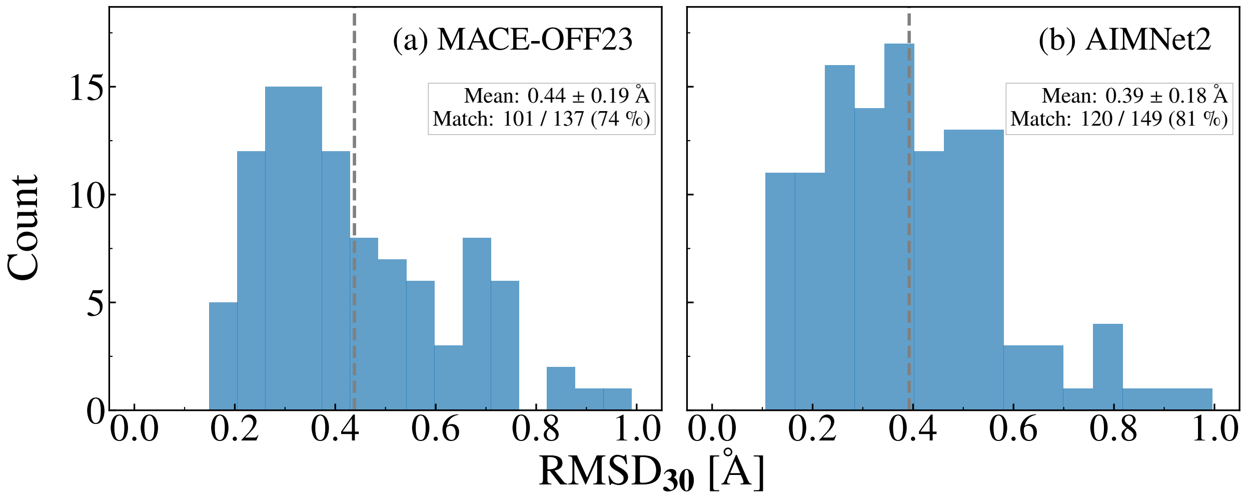


Figure S4: $RMSD_{30}$ histograms of the relaxed crystal structures obtained with the (a) MACE-OFF23(L) model and (b) system-specific AIMNet2 models compared to those obtained with PBE+MBD, starting from the same initial configuration, for $\varepsilon$-CL-20.

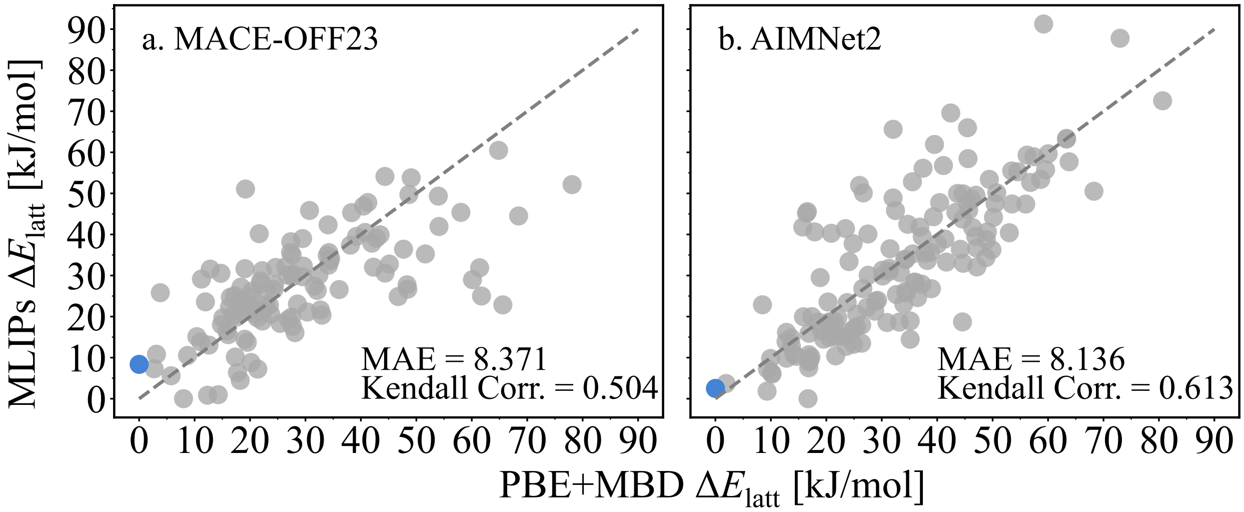


Figure S5: Relative lattice energies $\Delta E_{latt}$ obtained with the MACE-OFF23(L) model and system-specific AIMNet2, compared to those calculated using PBE+MBD for $\varepsilon$-CL-20. The experimentally observed structures are indicated in color. The mean absolute error (MAE), and Kendall correlation score are also shown.

# CSP results

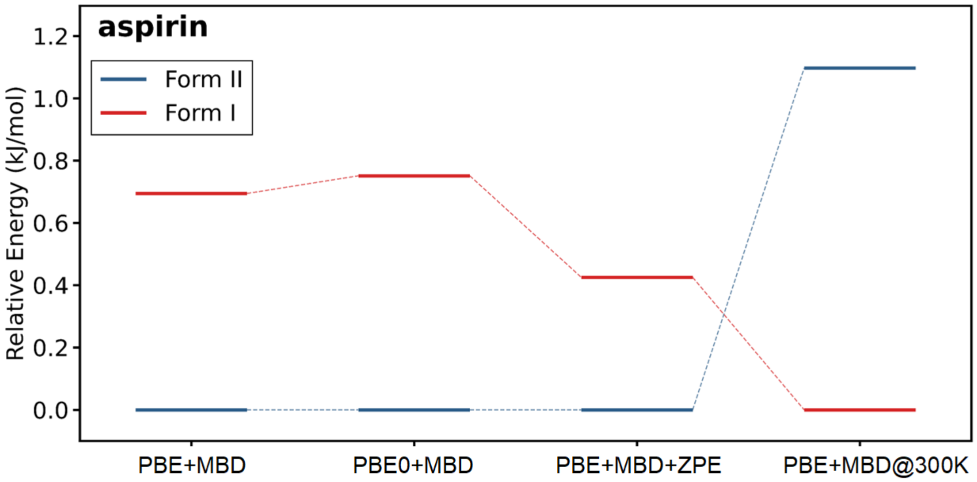


Figure S6: Relative stability ranking of aspirin Form I (red) and Form II (blue) computed with increasingly accurate DFT methods, and inclusion of zero-point energy (ZPE) corrections, followed by thermal effects at 300 K obtained using the quasi-harmonic approximation (QHA). Form I becomes more stable than Form II only when the free energy at room temperature is considered.

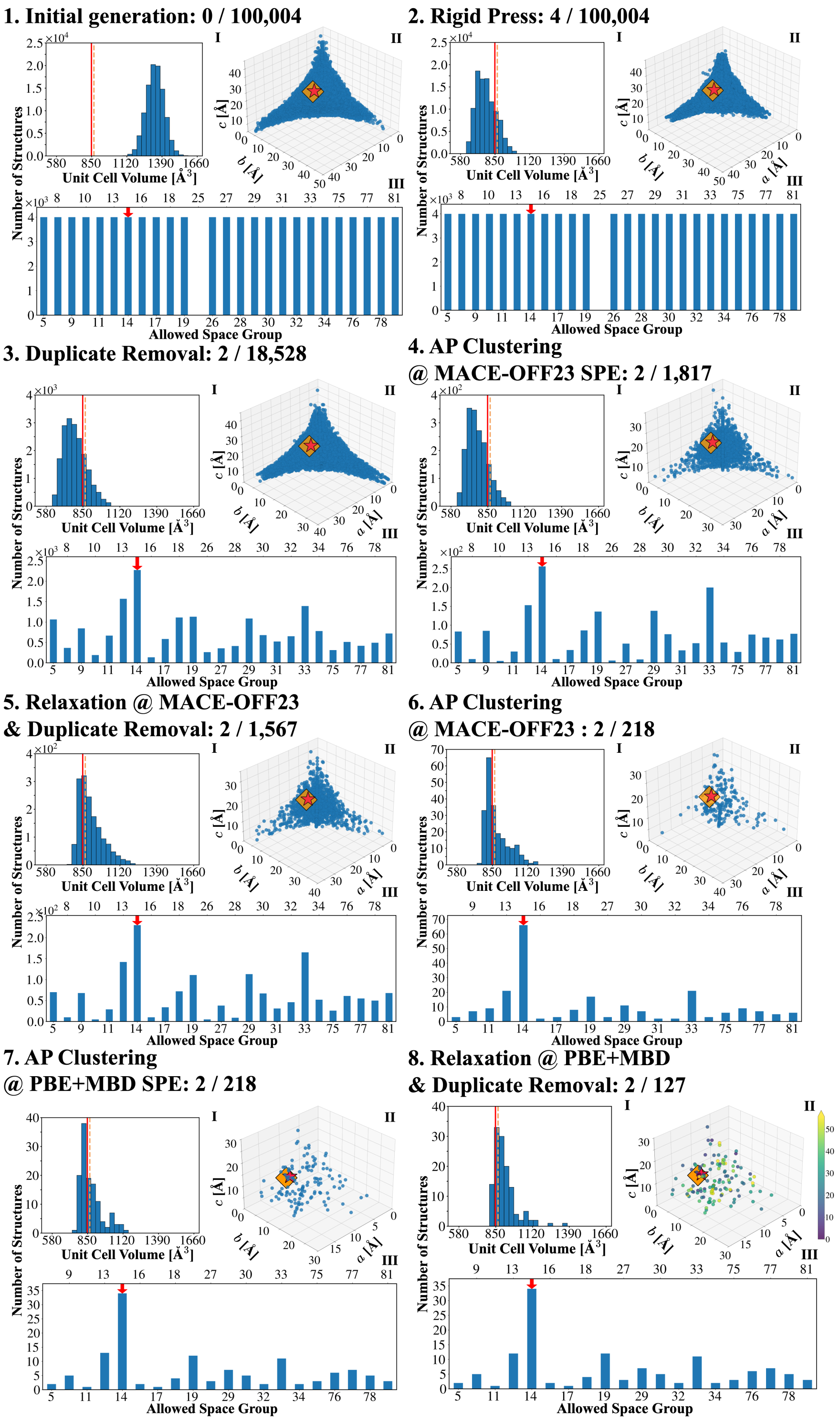


Figure S7: Distributions of unit cell volume, lattice parameters, and space groups, obtained at each step of the Genarris 3.0 workflow for aspirin with $Z = 4$. The experimental unit cell volume of Form I is indicated by a solid vertical red line and Form II is indicated by a dashed vertical orange line. The experimental lattice parameters of Form I and Form II are indicated by a red star and an orange square, respectively, and the experimental space group is indicated by a red arrow.

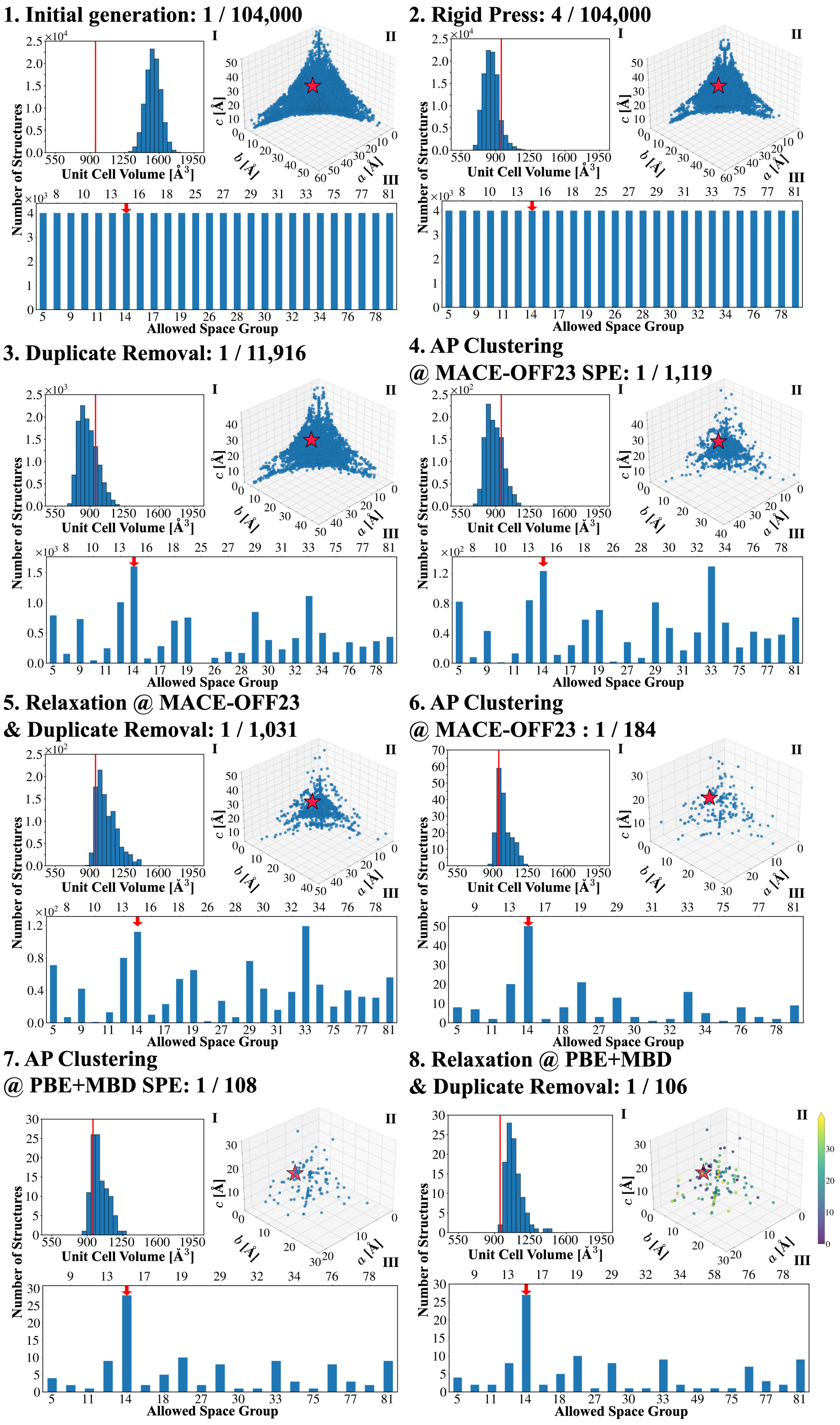


Figure S8: Distributions of unit cell volume, lattice parameters, and space groups, obtained at each step of the Genarris 3.0 workflow for Target XXII with $Z = 4$. The experimental unit cell volume is indicated by a solid vertical red line, the experimental lattice parameters are indicated by a red star, and the experimental space group is indicated by a red arrow.

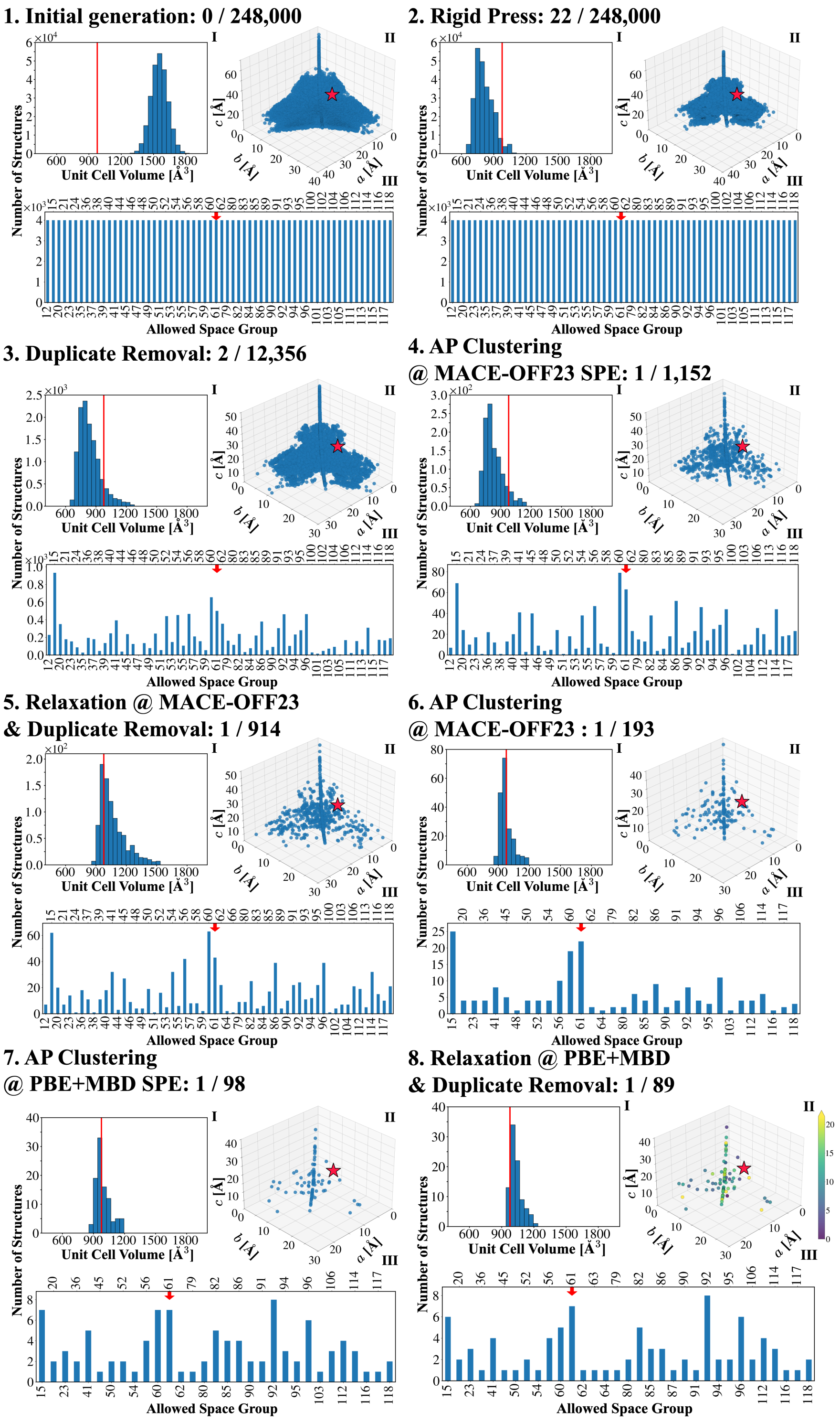


Figure S9: Distributions of unit cell volume, lattice parameters, and space groups, obtained at each step of the Genarris 3.0 workflow for Target I with $Z = 8$. The experimental unit cell volume is indicated by a solid vertical red line, the experimental lattice parameters are indicated by a red star, and the experimental space group is indicated by a red arrow. We note that structures with a large $c$ parameter are frequently generated because tetragonal crystal systems compatible with $Z = 8$, particularly space groups $P4_122$ and $P4_322$, inherently accommodate extended molecular arrangements along the $c$-axis.

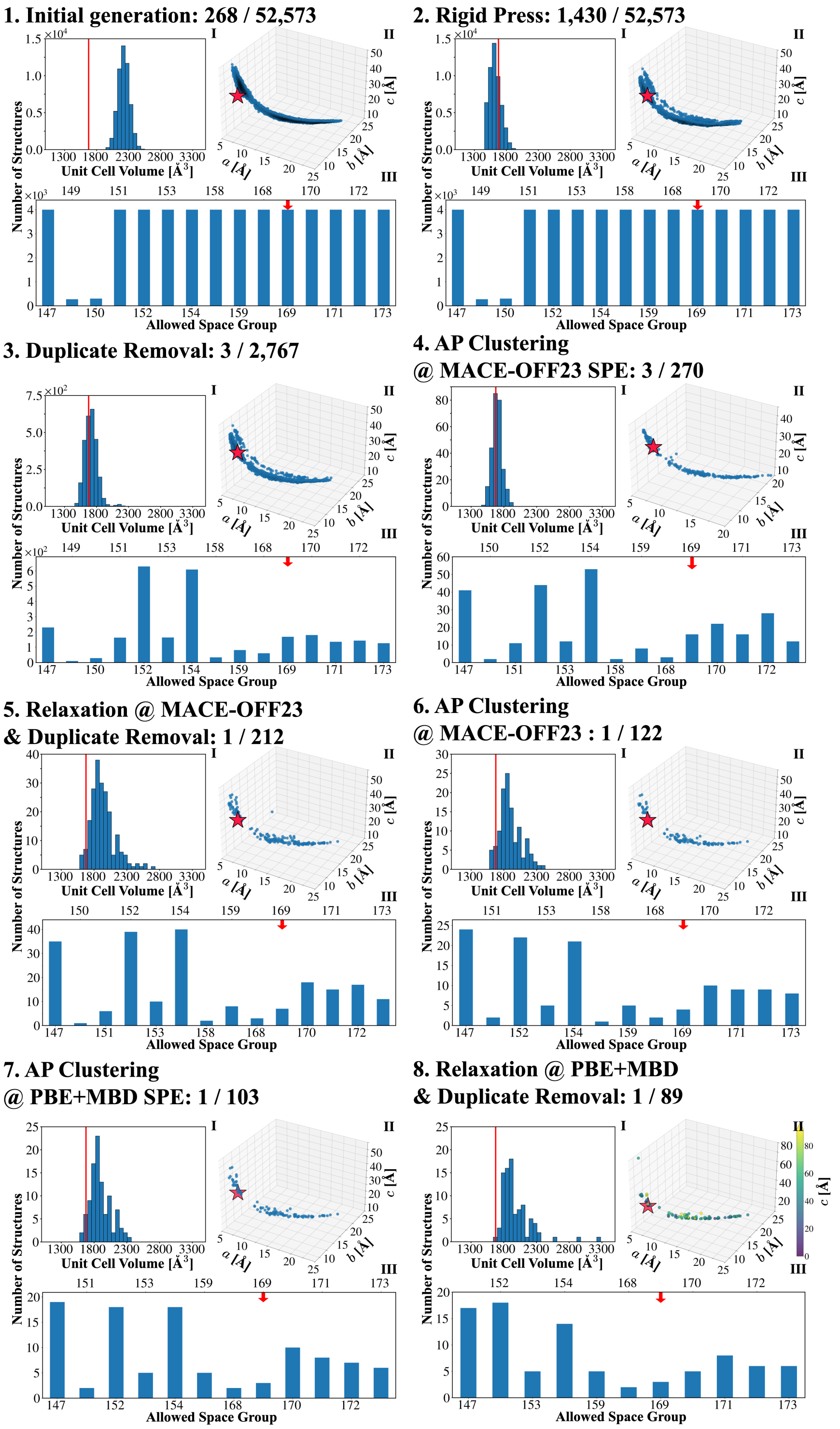


Figure S10: Distributions of unit cell volume, lattice parameters, and space groups, obtained at each step of the Genarris 3.0 workflow for $\delta$-HMX with $Z = 6$. The experimental unit cell volume is indicated by a vertical red line, the experimental lattice parameters are indicated by a red star, and the experimental space group is indicated by a red arrow.

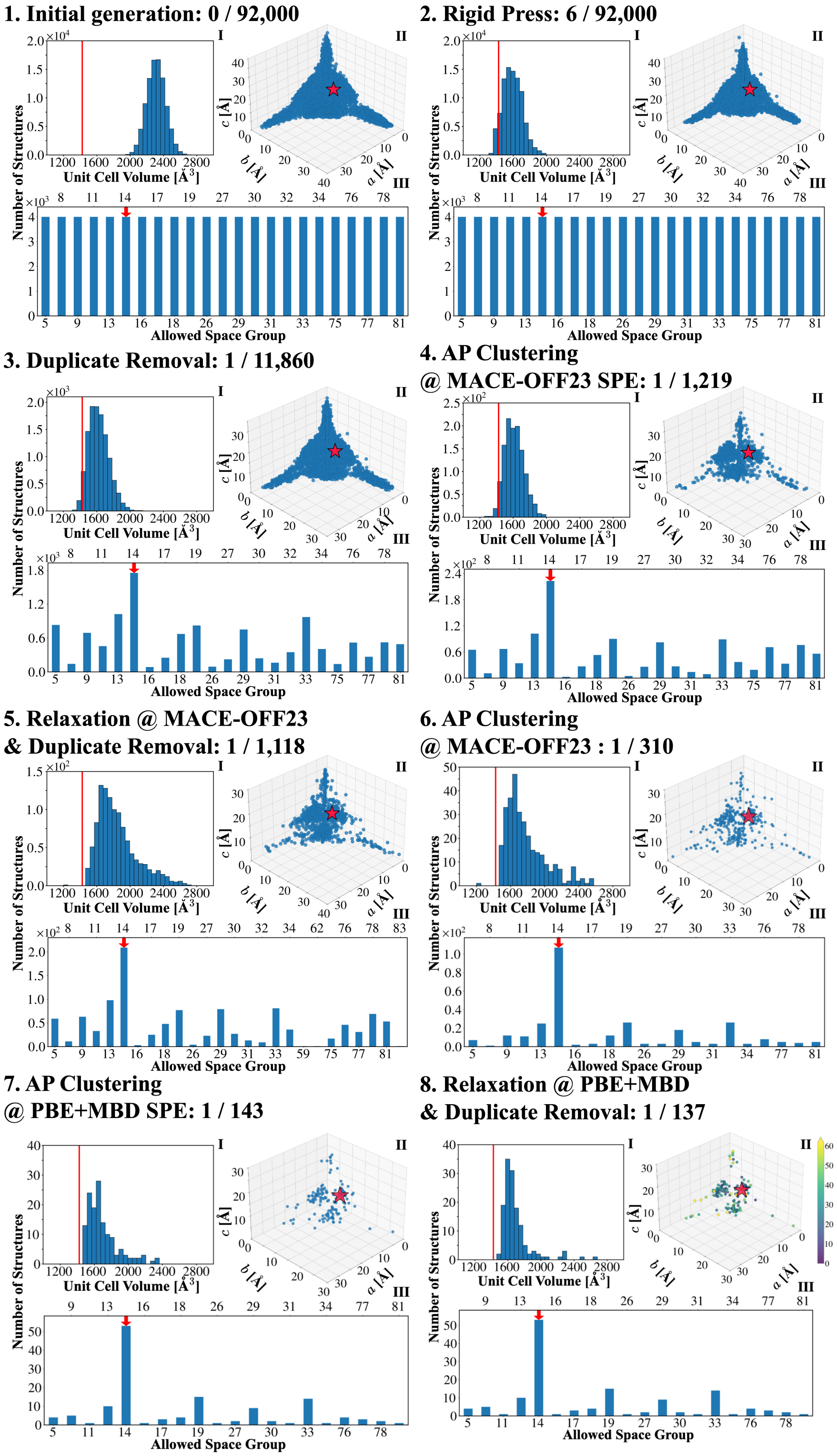


Figure S11: Distributions of unit cell volume, lattice parameters, and space groups, obtained at each step of the Genarris 3.0 workflow for $\varepsilon$-CL-20 with $Z = 4$. The experimental unit cell volume is indicated by a vertical red line, the experimental lattice parameters are indicated by a red star, and the experimental space group is indicated by a red arrow.

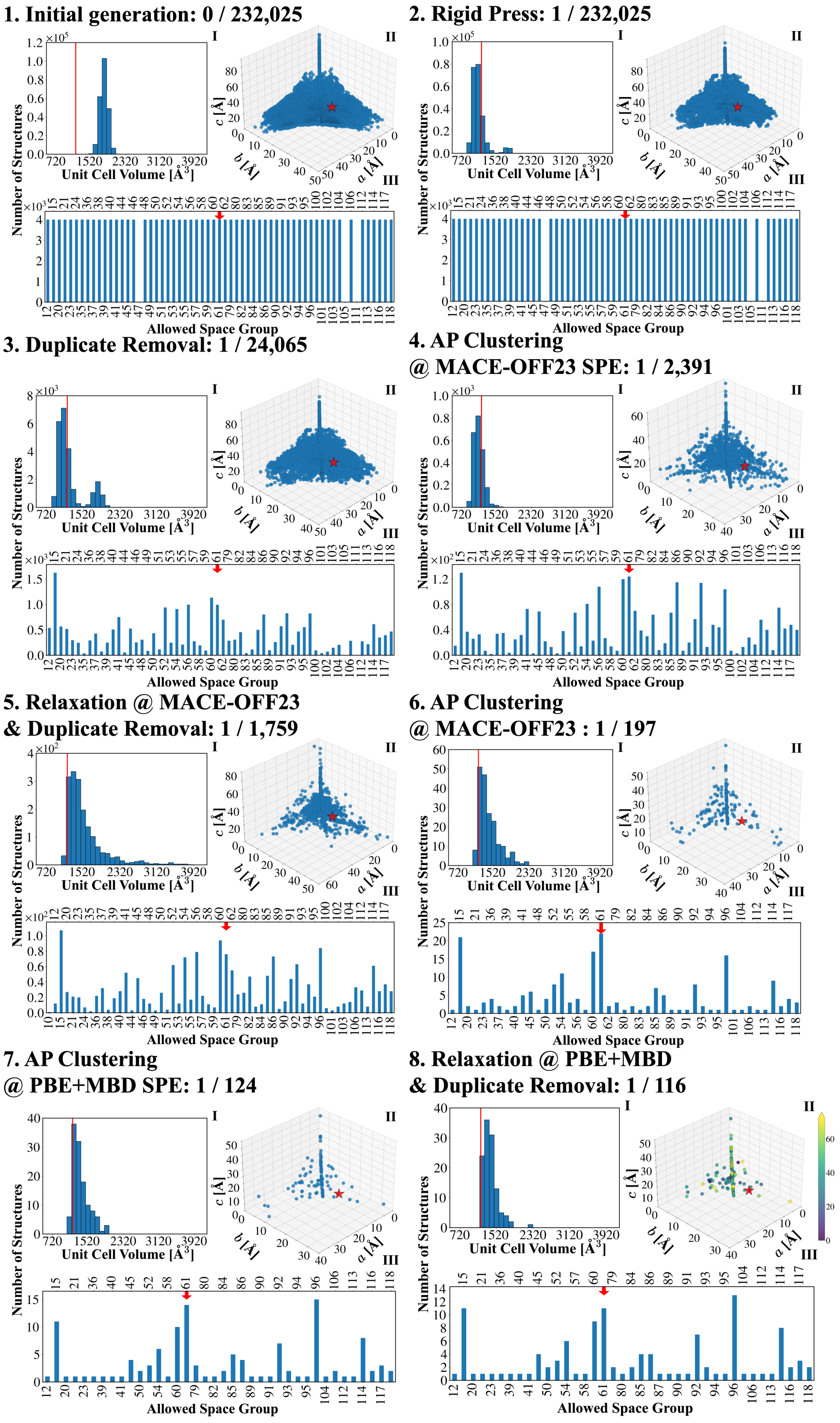


Figure S12: Distributions of unit cell volume, lattice parameters, and space groups, obtained at each step of the Genarris 3.0 workflow for DNI with $Z = 8$. The experimental unit cell volume is indicated by a solid vertical red line, the experimental lattice parameters are indicated by a red star, and the experimental space group is indicated by a red arrow. We note that structures with a large $c$ parameter are frequently generated because tetragonal crystal systems compatible with $Z = 8$, particularly space groups $P4_122$ and $P4_322$, inherently accommodate extended molecular arrangements along the $c$-axis.

# MACE-OFF Performance

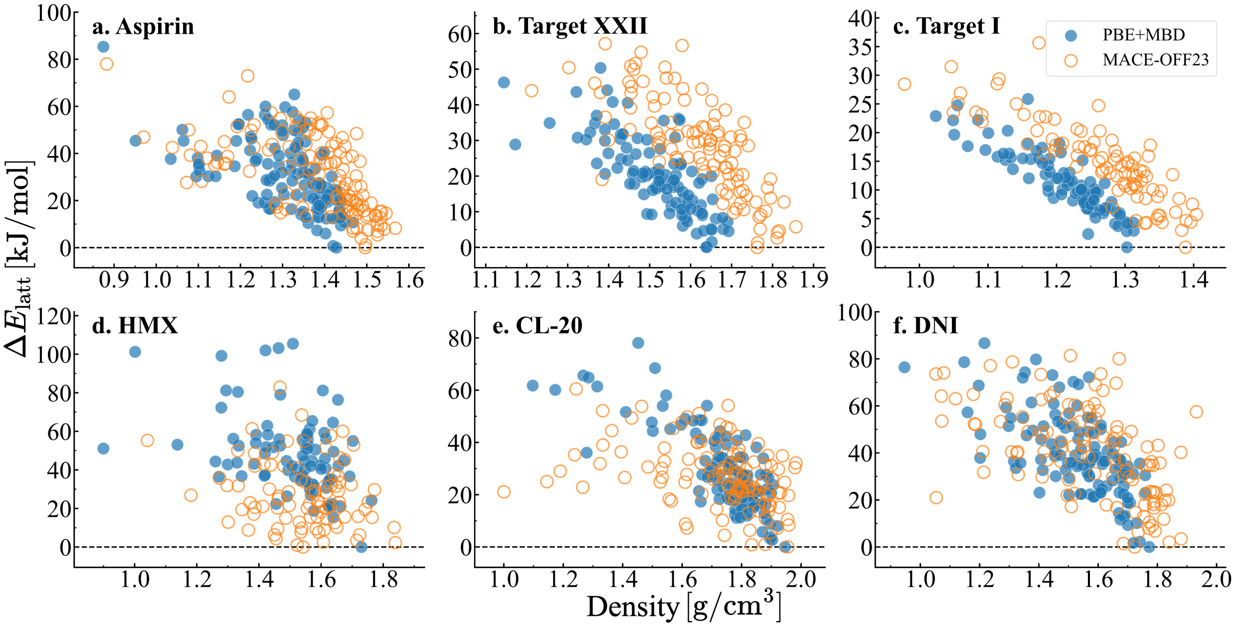


Figure S13: Energy landscapes for obtained using MACE-OFF23(L), shown in orange, compared to PBE+MBD, shown in blue, for the six CSP targets: (a) Aspirin, (b) Target XXII, (c) Target I, (d) HMX, (e) CL-20, and (f) DNI. For aspirin, Target XXII, and Target I the MACE-OFF23(L) landscapes are similar to the PBE+MBD landscapes but systematically shifted to higher densities. For the three energetic materials the MACE-OFF23(L) do not resemble the PBE+MBD landscapes as closely.

Table S3: Comparison of the structures obtained using MACE-OFF23(L) and PBE+MBD to the experimental structures of all targets in terms of lattice parameters, angles, unit cell volumes, and $RMSD_{30}$.

| **Polymorphs** | | $a$ (Å) | $b$ (Å) | $c$ (Å) | $\alpha$ (°) | $\beta$ (°) | $\gamma$ (°) | $V$ (Å$^3$) | $RMSD_{30}$ (Å) |
|---|---|---|---|---|---|---|---|---|---|
| **Aspirin Form I** | EXP | 11.446 | 6.596 | 11.388 | 90.00 | 95.55 | 90.00 | 855.74 | — |
| | MACE-OFF23(L) | 11.550 | 6.212 | 11.157 | 90.00 | 93.35 | 90.00 | 799.17 | 0.424 |
| | PBE+MBD | 11.721 | 6.328 | 11.391 | 90.00 | 94.27 | 90.00 | 842.49 | 0.295 |
| **Aspirin Form II** | EXP | 12.270 | 6.558 | 11.496 | 90.00 | 68.16 | 90.00 | 858.58 | — |
| | MACE-OFF23(L) | 12.045 | 6.344 | 11.308 | 90.00 | 67.76 | 90.00 | 799.80 | 0.261 |
| | PBE+MBD | 12.211 | 6.435 | 11.474 | 90.00 | 68.34 | 90.00 | 838.03 | 0.131 |
| **Target XXII** | EXP | 11.947 | 6.696 | 12.598 | 90.00 | 108.60 | 90.00 | 955.16 | — |
| | MACE-OFF23(L) | 11.419 | 6.675 | 14.727 | 90.00 | 123.58 | 90.00 | 935.11 | 0.362 |
| | PBE+MBD | 12.057 | 6.816 | 14.447 | 90.00 | 122.01 | 90.00 | 1006.65 | 0.176 |
| **Target I** | EXP | 5.309 | 12.648 | 14.544 | 90.00 | 90.00 | 90.00 | 976.60 | — |
| | MACE-OFF23(L) | 5.412 | 12.319 | 13.509 | 90.00 | 90.00 | 90.00 | 900.57 | 0.671 |
| | PBE+MBD | 5.351 | 12.563 | 14.276 | 90.00 | 90.00 | 90.00 | 959.60 | 0.710 |
| **$\delta$-HMX** | EXP | 7.711 | 7.711 | 32.553 | 90.00 | 90.00 | 120.00 | 1676.27 | — |
| | MACE-OFF23(L) | 7.742 | 7.742 | 32.465 | 90.00 | 90.00 | 120.00 | 1685.41 | 0.193 |
| | PBE+MBD | 7.768 | 7.768 | 32.768 | 90.00 | 90.00 | 120.00 | 1712.53 | 0.135 |
| **$\varepsilon$-CL-20** | EXP | 8.852 | 12.556 | 13.386 | 90.00 | 106.82 | 90.00 | 1424.10 | — |
| | MACE-OFF23(L) | 8.894 | 12.718 | 14.108 | 90.00 | 68.61 | 90.00 | 1485.80 | 0.286 |
| | PBE+MBD | 8.930 | 12.773 | 14.089 | 90.00 | 68.51 | 90.00 | 1495.39 | 0.211 |
| **24DNI** | EXP | 10.127 | 18.497 | 6.333 | 90.00 | 90.00 | 90.00 | 1186.29 | — |
| | MACE-OFF23(L) | 10.318 | 18.171 | 6.258 | 90.00 | 90.00 | 90.00 | 1173.24 | 0.259 |
| | PBE+MBD | 10.018 | 18.608 | 6.354 | 90.00 | 90.00 | 90.00 | 1184.55 | 0.095 |

Figure S14 presents an analysis of the correlation between the relaxation performance of MACE-OFF23(L) and the relative lattice energy of putative molecular crystal structures. The analysis was performed in order to test whether MACE-OFF23(L) performs better for structures that are more reasonable. $RMSD_{30}$ values were calculated by comparing crystal structures optimized using the MACE-OFF23(L) against reference structures optimized using PBE+MBD. Then, single-point energy (SPE) evaluations with PBE+MBD were performed on the MACE-OFF23(L) relaxed structures to determine their relative lattice energies with respect to the minimum energy structure for each target.

The box plots on the left (Figure S14 panels i) display the distribution of $RMSD_{30}$ values grouped by relative lattice energy quartiles, where Q1 represents the most thermodynamically stable structures (lowest relative energies) and Q4 represents the least stable structures (highest relative energies).

The performance trend plots on the right (Figure S14 panels ii) were constructed by binning structures according to their relative lattice energies. The relative energy range for each target was divided into 10 equally spaced bins and the mean $RMSD_{30}$ value was calculated for all structures falling within each energy bin. Error bars represent the standard deviation of $RMSD_{30}$ values within each bin, shown as shaded bands around the mean trend line. Linear trend lines (dashed gray lines) were fitted to illustrate the overall correlation between relative lattice energy and structure similarity.

Overall, we find that in most cases the MACE-OFF23(L) relaxation performance is weakly correlated with relative stability. However, for $\delta$-HMX it is inversely correlated.

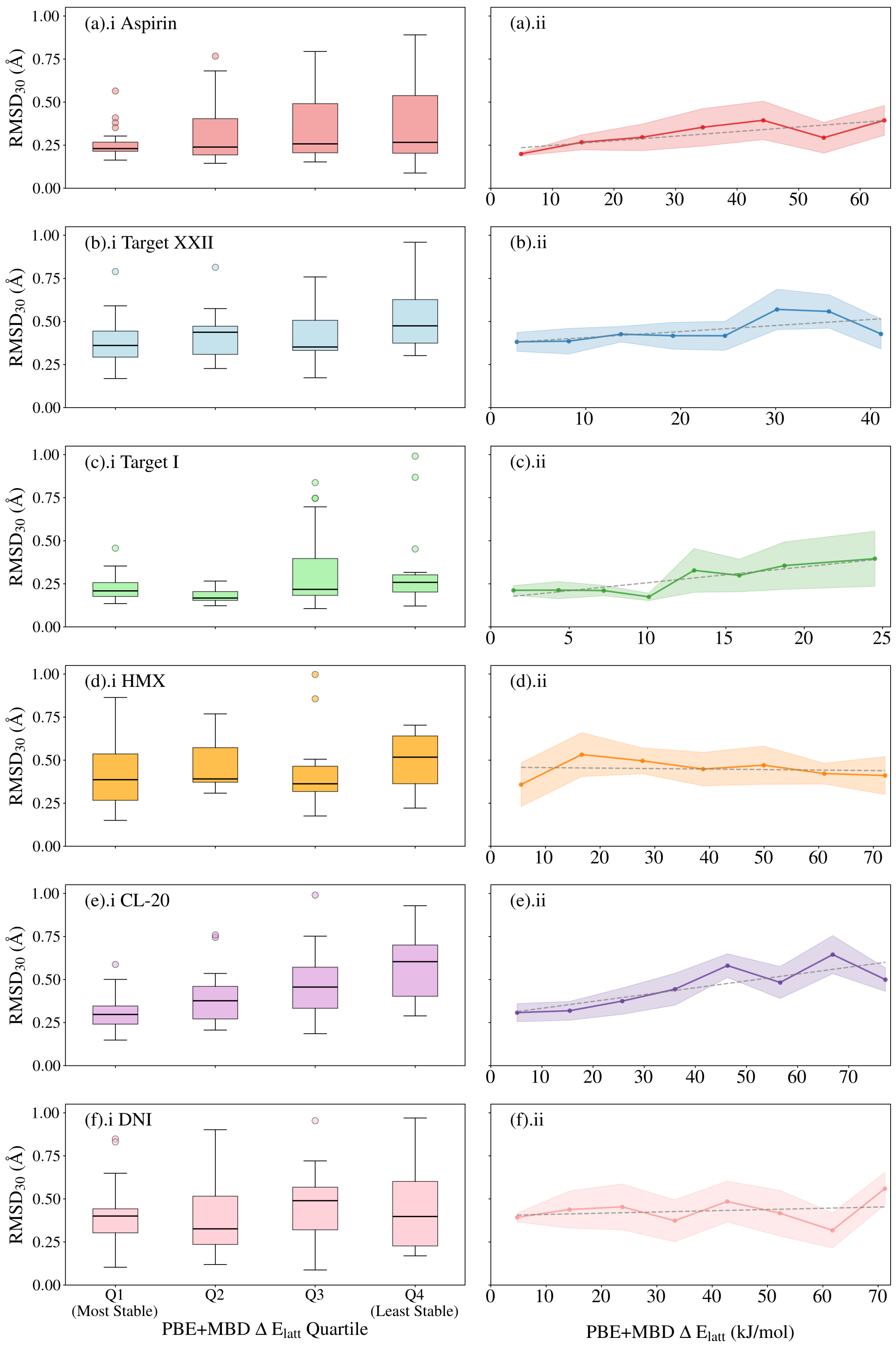


Figure S14: Correlation between MACE-OFF23(L) relaxation performance and the relative lattice energy of putative molecular crystal structures. (i) Distribution of $\mathrm{RMSD}_{30}$ values comparing MACE-OFF23(L) optimized structures to reference PBE+MBD optimized structures, grouped by relative lattice energy quartiles (Q1: most stable, Q4: least stable). (ii) Mean $\mathrm{RMSD}_{30}$ versus relative lattice energy, with standard deviation shown as shaded bands and linear trends indicated by dashed gray lines.

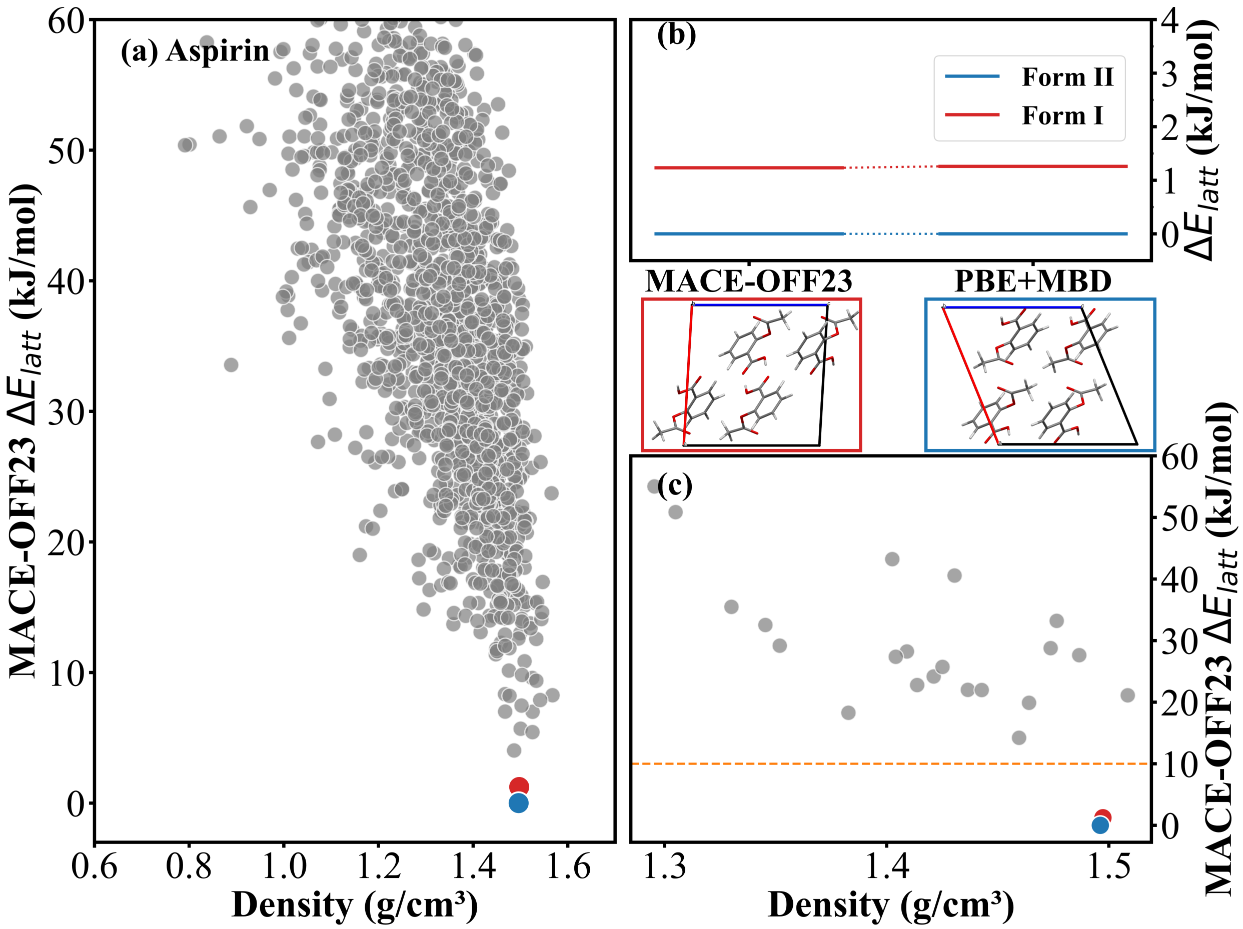


Figure S15: The clustering and down-selection workflow for aspirin. (a) Relative lattice energies computed using MACE-OFF23(L) as a function of crystal density after relaxation with MACE-OFF23(L). The predicted experimental structures Form I (red) and Form II (blue) are highlighted. (b) Comparison of relative lattice energies computed with MACE-OFF23(L) and PBE+MBD for the two structures, which are also shown. The PBE+MBD calculations were performed on the structures relaxed with MACE-OFF23(L). (c) MACE-OFF23(L) relative energy as a function of crystal density for the cluster containing the experimental structures. The orange dashed line indicates the 10 kJ/mol energy threshold. For aspirin, the two experimental forms are ranked by MACE-OFF23(L) as the most stable overall. Both forms are grouped together in the same cluster. Because an energy window is applied for down-selection, both structures proceed to the next steps in the workflow.

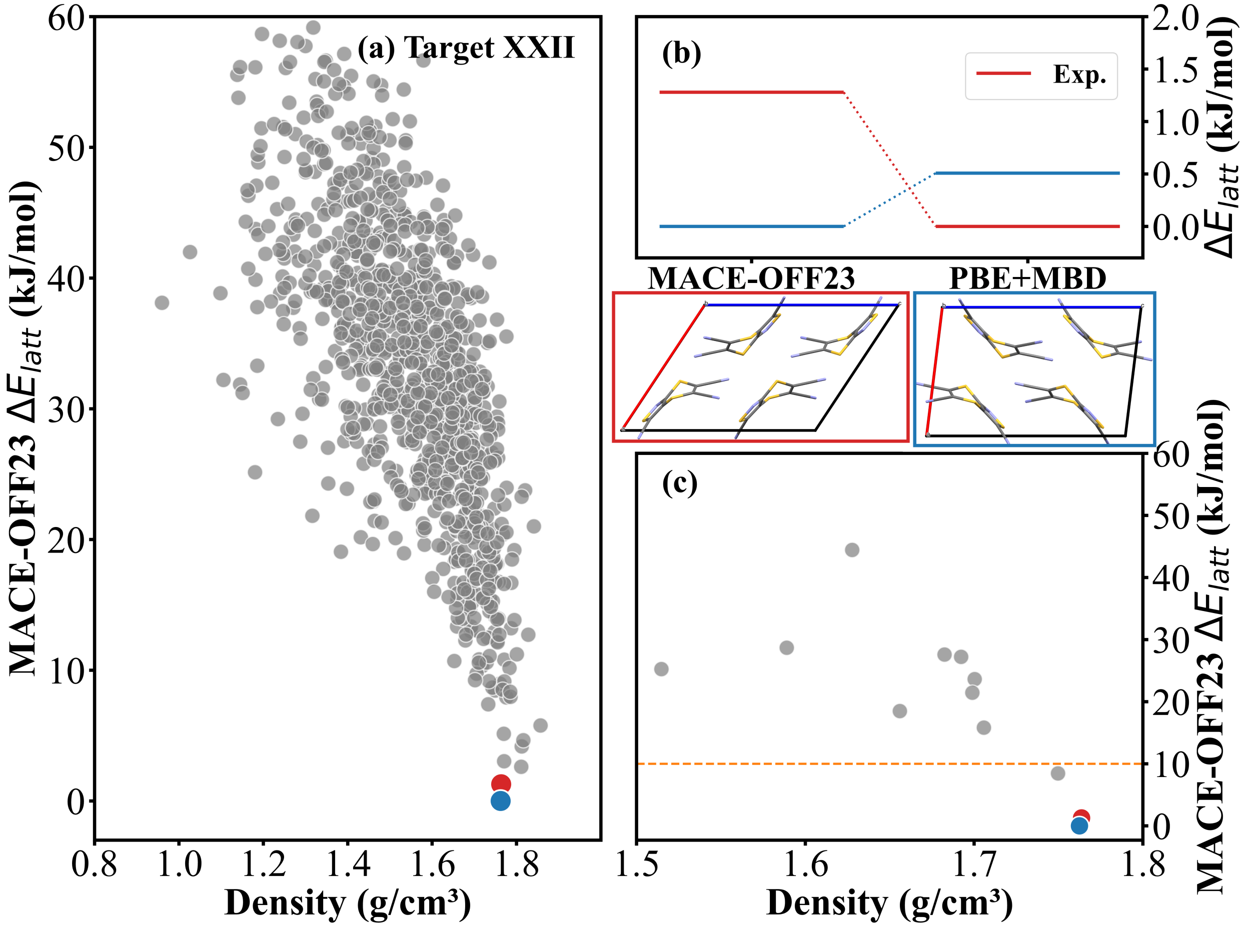


Figure S16: The clustering and down-selection workflow for Target XXII. (a)Relative lattice energies computed using MACE-OFF23(L) as a function of crystal density after relaxation with MACE-OFF23(L). The experimental structure (red) and the MACE-OFF23(L) lowest energy structure (blue) are highlighted. (b) Comparison of relative lattice energies computed with MACE-OFF23(L) and PBE+MBD for the two structures, which are also shown. The PBE+MBD calculations were performed on the structures relaxed with MACE-OFF23(L). (c) MACE-OFF23(L) relative energy as a function of crystal density for the cluster containing the experimental structure. The orange dashed line indicates the 10 kJ/mol energy threshold. The experimental structure is ranked by MACE-OFF23(L) as the second lowest in energy overall, after the structure colored in blue. Both structures are grouped together in the same cluster. Because an energy window is applied for down-selection, both structures proceed to the next steps in the workflow.

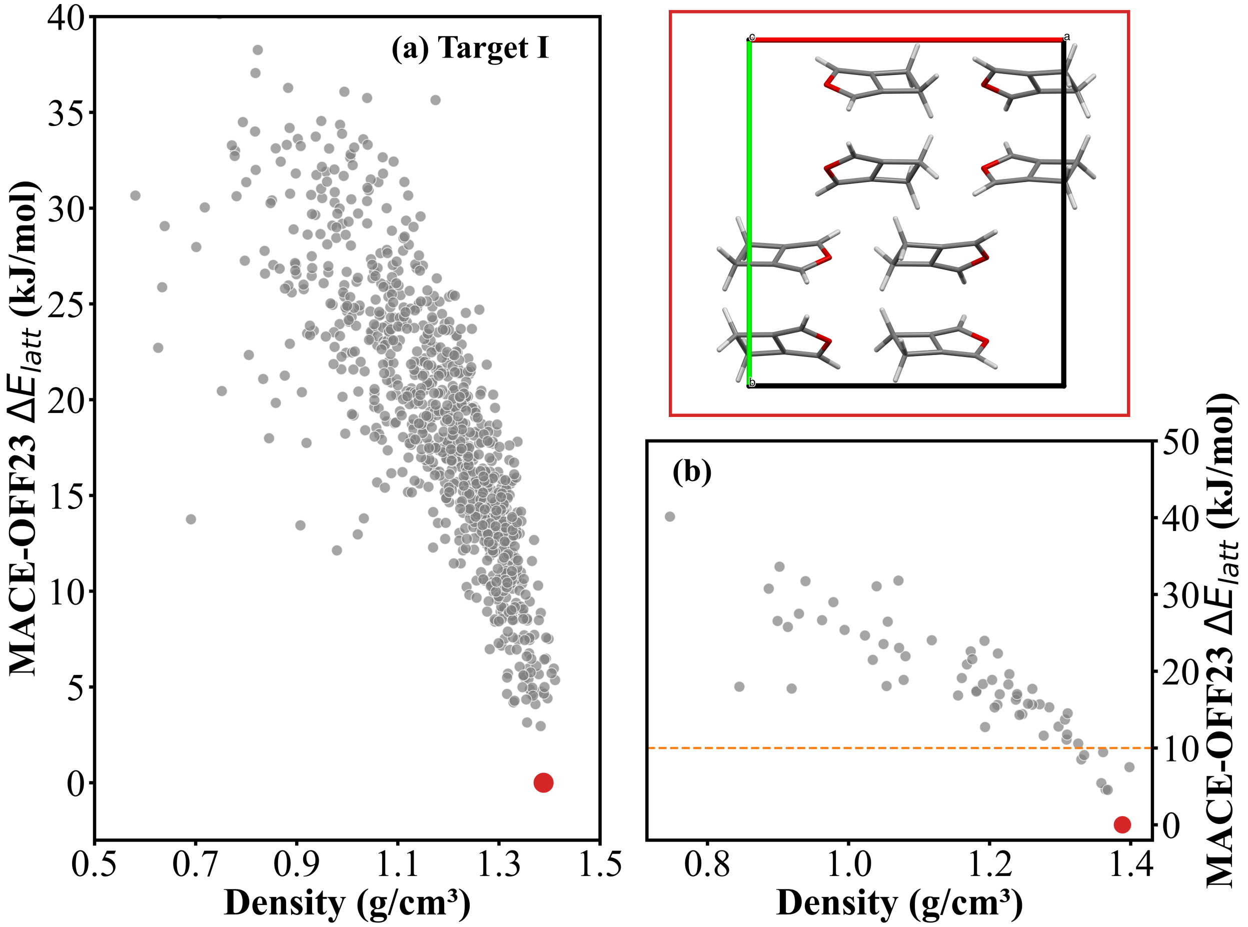


Figure S17: The clustering and down-selection workflow for Target I. (a) Relative lattice energies computed using MACE-OFF23(L) as a function of crystal density after relaxation with MACE-OFF23(L). (b) MACE-OFF23(L) relative energy as a function of crystal density for the cluster containing the experimental structure. The orange dashed line indicates the 10 kJ/mol energy threshold. The experimental structure is ranked as the lowest energy structure overall and in its cluster. It is selected along with 4 other putative structures to proceed to the next steps of the workflow.

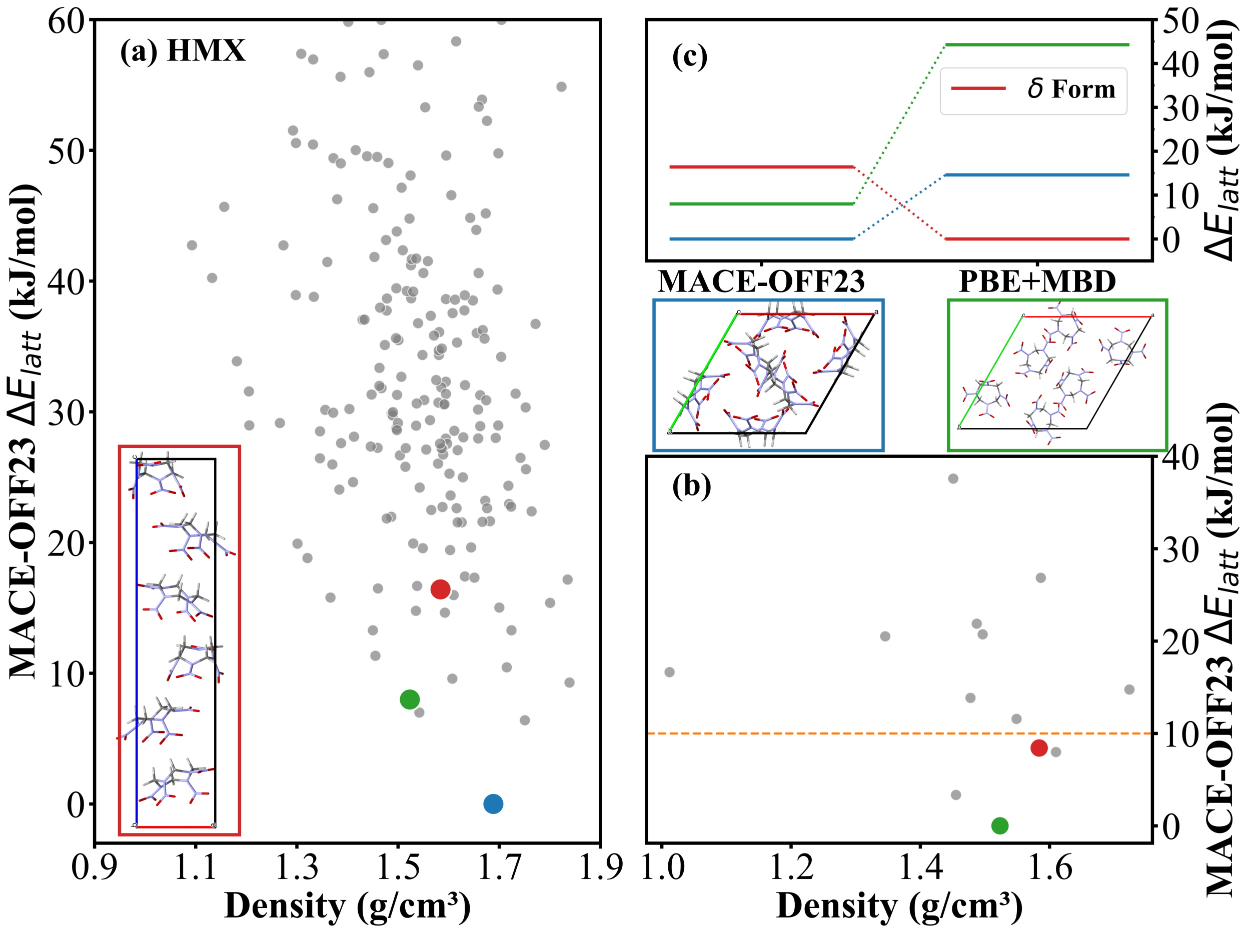


Figure S18: The clustering and down-selection workflow for $\delta$-HMX. (a) Relative lattice energies computed using MACE-OFF23(L) as a function of crystal density after relaxation with MACE-OFF23(L). The experimental structure (red), the MACE-OFF23(L) lowest energy structure (blue) and the lowest energy structure in the cluster containing the experimental structure (green) are highlighted. (b) Comparison of relative lattice energies computed with MACE-OFF23(L) and PBE+MBD for the three structures, which are also shown. The PBE+MBD calculations were performed on the structures relaxed with MACE-OFF23(L). (c) MACE-OFF23(L) relative energy as a function of crystal density for the cluster containing the experimental structure. The orange dashed line indicates the 10 kJ/mol energy threshold. Similar to the case of DNI, shown in the main text, the experimental structure is ranked poorly by MACE-OFF23(L), close to 20 kJ/mol above the global minimum. Even in its cluster, the experimental structure is ranked as #4, close to 10 kJ/mol above the structure shown in green, but it till passes the selection threshold and proceeds to the next steps of the workflow.

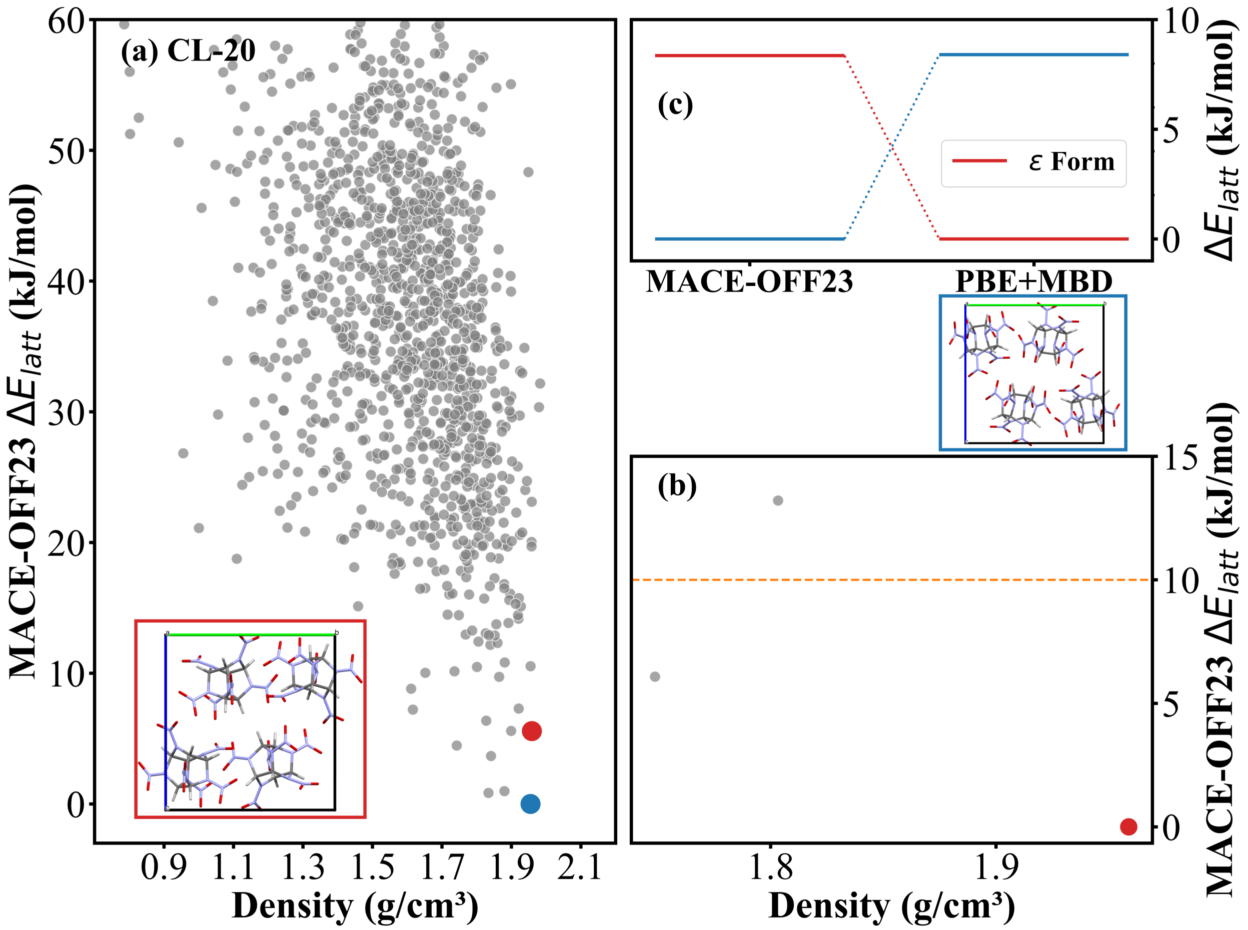


Figure S19: The clustering and down-selection workflow for $\varepsilon$-CL-20. (a) (a) Relative lattice energies computed using MACE-OFF23(L) as a function of crystal density after relaxation with MACE-OFF23(L). The experimental structure (red) and the MACE-OFF23(L) lowest energy structure (blue) are highlighted. (b) Comparison of relative lattice energies computed with MACE-OFF23(L) and PBE+MBD for the two structures, which are also shown. The PBE+MBD calculations were performed on the structures relaxed with MACE-OFF23(L). (c) MACE-OFF23(L) relative energy as a function of crystal density for the cluster containing the experimental structure. The orange dashed line indicates the 10 kJ/mol energy threshold. The experimental structure is misranked by MACE-OFF23(L), however it has the lowest energy in its cluster and therefore it is selected to proceed to the next steps of the workflow.

# References

(1) Spicher, S.; Grimme, S. Robust atomistic modeling of materials, organometallic, and biochemical systems. *Angewandte Chemie International Edition* **2020**, *59*, 15665–15673.

(2) Bannwarth, C.; Ehlert, S.; Grimme, S. GFN2-xTB—An accurate and broadly parametrized self-consistent tight-binding quantum chemical method with multipole electrostatics and density-dependent dispersion contributions. *Journal of Chemical Theory and Computation* **2019**, *15*, 1652–1671.

(3) Weigend, F.; Ahlrichs, R. Balanced basis sets of split valence, triple zeta valence and quadruple zeta valence quality for H to Rn: Design and assessment of accuracy. *Physical Chemistry Chemical Physics* **2005**, *7*, 3297–3305.

(4) Weigend, F. Accurate Coulomb-fitting basis sets for H to Rn. *Physical Chemistry Chemical Physics* **2006**, *8*, 1057–1065.

(5) Neese, F. Software update: The ORCA program system—Version 5.0. *Wiley Interdisciplinary Reviews: Computational Molecular Science* **2022**, *12*, e1606.

(6) Neese, F. Software update: The ORCA program system—version 6.0. *Wiley Interdisciplinary Reviews: Computational Molecular Science* **2025**, *15*, e70019.

(7) Grimme, S.; Ehrlich, S.; Goerigk, L. Effect of the damping function in dispersion corrected density functional theory. *Journal of Computational Chemistry* **2011**, *32*, 1456–1465.

(8) Nayal, K. S.; O'Connor, D.; Zubatyuk, R.; Anstine, D. M.; Yang, Y.; Tom, R.; Deng, W.; Tang, K.; Marom, N.; Isayev, O. Efficient Molecular Crystal Structure Prediction and Stability Assessment with AIMNet2 Neural Network Potentials. *ChemRxiv* **2025**, DOI: 10.26434/chemrxiv–2025–ksn4n.
